# Stability of noisy Metropolis–Hastings

**DOI:** 10.1007/s11222-015-9604-3

**Published:** 2015-11-07

**Authors:** F. J. Medina-Aguayo, A. Lee, G. O. Roberts

**Affiliations:** Department of Statistics, University of Warwick, Coventry, CV4 7AL UK

**Keywords:** Markov chain Monte Carlo, Pseudo-marginal Monte Carlo, Monte Carlo within Metropolis, Intractable likelihoods, Geometric ergodicity

## Abstract

Pseudo-marginal Markov chain Monte Carlo methods for sampling from intractable distributions have gained recent interest and have been theoretically studied in considerable depth. Their main appeal is that they are exact, in the sense that they target marginally the correct invariant distribution. However, the pseudo-marginal Markov chain can exhibit poor mixing and slow convergence towards its target. As an alternative, a subtly different Markov chain can be simulated, where better mixing is possible but the exactness property is sacrificed. This is the noisy algorithm, initially conceptualised as Monte Carlo within Metropolis, which has also been studied but to a lesser extent. The present article provides a further characterisation of the noisy algorithm, with a focus on fundamental stability properties like positive recurrence and geometric ergodicity. Sufficient conditions for inheriting geometric ergodicity from a standard Metropolis–Hastings chain are given, as well as convergence of the invariant distribution towards the true target distribution.

**Electronic supplementary material:**

The online version of this article (doi:10.1007/s11222-015-9604-3) contains supplementary material, which is available to authorized users.

## Introduction

### Intractable target densities and the pseudo-marginal algorithm

Suppose our aim is to simulate from an intractable probability distribution $\pi$ for some random variable *X*, which takes values in a measurable space $\left( \mathcal {X},\mathcal {B}(\mathcal {X})\right)$. In addition, let $\pi$ have a density $\pi (x)$ with respect to some reference measure $\mu (dx)$, e.g. the counting or the Lebesgue measure. By intractable we mean that an analytical expression for the density $\pi (x)$ is not available and so implementation of a Markov chain Monte Carlo (MCMC) method targeting $\pi$ is not straightforward.

One possible solution to this problem is to target a different distribution on the extended space $\left( \mathcal {X}\times \mathcal {W},\mathcal {B}(\mathcal {X})\times \mathcal {B}(\mathcal {W})\right)$, which admits $\pi$ as marginal distribution. The pseudo-marginal algorithm (Beaumont 2003; Andrieu and Roberts 2009) falls into this category since it is a Metropolis–Hastings (MH) algorithm targeting a distribution $\bar{\pi }_{N}$, associated to the random vector (*X*, *W*) defined on the product space $\left( \mathcal {X}\times \mathcal {W},\mathcal {B}(\mathcal {X})\times \mathcal {B}(\mathcal {W})\right)$ where $\mathcal {W}\subseteq {\mathbb {R}}^{+}_0:=[0,\infty )$. It is given by

1 $$\begin{aligned} \bar{\pi }_{N}(dx,dw) := \pi (dx)Q_{x,N}(dw)w, \end{aligned}$$

where $\left\{ Q_{x,N}\right\} _{(x,N)\in \mathcal {X}\times {\mathbb {N}}^{+}}$ is a family of probability distributions on $\left( \mathcal {W},\mathcal {B}(\mathcal {W})\right)$ satisfying for each $(x,N)\in \mathcal {X}\times {\mathbb {N}}$

2 $$\begin{aligned} {\mathbb {E}}_{Q_{x,N}}\left[ W_{x,N}\right] \equiv 1,\quad \text {for}\;W_{x,N}\sim Q_{x,N}(\cdot ). \end{aligned}$$

Throughout this article, we restrict our attention to the case where for each $x\in \mathcal {X}$, $W_{x,N}$ is $Q_{x,N}$-a.s. strictly positive, for reasons that will become clear.

The random variables $\left\{ W_{x,N}\right\} _{x,N}$ are commonly referred as the weights. Formalising this algorithm using (1) and (2) was introduced by Andrieu and Vihola (2015), and “exactness” follows immediately: $\bar{\pi }$ admits $\pi$ as a marginal. Given a proposal kernel $q:\mathcal {X}\times \mathcal {B}(\mathcal {X})\rightarrow [0,1]$, the respective proposal of the pseudo-marginal is given by

$$\begin{aligned} {\bar{q}}_{N}(x,w;dy,du) := q(x,dy)Q_{y,N}(du), \end{aligned}$$

and, consequently, the acceptance probability can be expressed as

3 $$\begin{aligned} {\bar{\alpha }}_{N}(x,w;y,u) := \min \left\{ 1,\frac{\pi (dy)uq(y,dx)}{\pi (dx)wq(x,dy)}\right\} . \end{aligned}$$

The pseudo-marginal algorithm defines a time-homogeneous Markov chain, with transition kernel $\bar{P}_{N}$ on the measurable space $\left( \mathcal {X}\times \mathcal {W},\mathcal {B}(\mathcal {X})\times \mathcal {B}(\mathcal {W})\right)$. A single draw from $\bar{P}_{N}(x,w;\cdot ,\cdot )$ is presented in Algorithm 1.
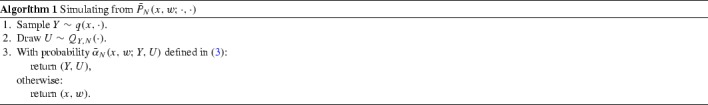


Due to its exactness and straightforward implementation in many settings, the pseudo-marginal has gained recent interest and has been theoretically studied in some depth, see e.g. Andrieu and Roberts (2009), Andrieu and Vihola (2014, 2015), Doucet et al. (2015), Girolami et al. (2013), Maire et al. (2014) and Sherlock et al. (2015). These studies typically compare the pseudo-marginal Markov chain with a “marginal” Markov chain, arising in the case where all the weights are almost surely equal to 1, and (3) is then the standard Metropolis–Hastings acceptance probability associated with the target density $\pi$ and the proposal *q*.

### Examples of pseudo-marginal algorithms

A common source of intractability for $\pi$ occurs when a latent variable *Z* on $(Z,\mathcal {B}(Z))$ is used to model observed data, as in hidden Markov models (HMMs) or mixture models. Although the density $\pi (x)$ cannot be computed, it can be approximated via importance sampling, using an appropriate auxiliary distribution, say $\nu _{x}$. Here, appropriate means $\pi _{x}\ll \nu _{x}$, where $\pi _{x}$ denotes the conditional distribution of *Z* given $X=x$. Therefore, for this setting, the weights are given by

$$\begin{aligned}&W_{x,N} = \frac{1}{N}\sum _{k=1}^{N}\frac{\pi _{x}\left( Z_{x}^{(k)}\right) }{\nu _{x}\left( Z_{x}^{(k)}\right) },\nonumber \\&\text {where}\;\left\{ Z_{x}^{(k)}\right\} _{k\in \{1,\ldots ,N\}}\overset{i.i.d.}{\sim }\nu _{x}(\cdot ), \end{aligned}$$

which motivates the following generic form when using averages of unbiased estimators

4 $$\begin{aligned}&W_{x,N} = \frac{1}{N}\sum _{k=1}^{N}W_{x}^{(k)},\nonumber \\&\text {where}\;\left\{ W_{x}^{(k)}\right\} _{k}\overset{i.i.d.}{\sim }Q_{x}(\cdot ),{\mathbb {E}}_{Q_{x}}\left[ W_{x}^{(k)}\right] \equiv 1. \end{aligned}$$

It is clear that (4) describes only a special case of (2). Nevertheless, we will pay special attention to the former throughout the article. For similar settings to (4) see Andrieu and Roberts (2009).

**Fig. 1:**
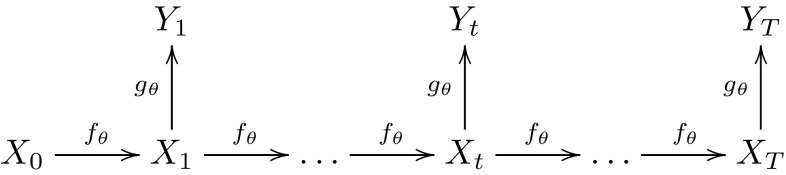
Hidden Markov model

Since (2) is more general, it allows $W_{x,N}$ to be any random variable with expectation 1. Sequential Monte Carlo (SMC) methods involve the simulation of a system of some number of particles, and provide unbiased estimates of likelihoods associated with HMMs (see Del Moral 2004, Proposition 7.4.1 or Pitt et al. 2012) irrespective of the size of the particle system. Consider the model given by Fig. 1. The random variables $\left\{ X_{t}\right\} _{t=0}^{T}$ form a time-homogeneous Markov chain with transition $f_{\theta }(\cdot |x_{t-1})$ that depends on a set of parameters $\theta$. The observed random variables $\left\{ Y_{t}\right\} _{t=1}^{T}$ are conditionally independent given the unobserved $\left\{ X_{t}\right\} _{t=1}^{T}$ and are distributed according to $g_{\theta }(\cdot |x_{t})$, which also may depend on $\theta$. The likelihood function for $\theta$ is given by

$$\begin{aligned} l(\theta ;y_1,\ldots ,y_T):= {\mathbb {E}}_{f_\theta } \left[ \prod _{t=1}^T g_\theta (y_{t}|X_{t}) \right] , \end{aligned}$$

where ${{\mathbb {E}}_f}_\theta$ denotes expectation w.r.t. the $\theta$-dependent law of $\{X_t\}_{t=1}^T$, and we assume for simplicity that the initial value $X_0=x_0$ is known. If we denote by $\hat{l}_N(\theta ;y_1,\ldots ,y_T)$ the unbiased SMC estimator of $l(\theta ;y_1,\ldots ,y_T)$ based on *N* particles, we can then define

$$\begin{aligned} W_{\theta ,N}:= \frac{\hat{l}_N(\theta ;y_1,\ldots ,y_T)}{l(\theta ;y_1,\ldots ,y_T)}, \end{aligned}$$

and (2) is satisfied but (4) is not. The resulting pseudo-marginal algorithm is developed and discussed in detail in Andrieu et al. (2010), where it and related algorithms are referred to as particle MCMC methods.

### The noisy algorithm

Although the pseudo-marginal has the desirable property of exactness, it can suffer from “sticky” behaviour, exhibiting poor mixing and slow convergence towards the target distribution (Andrieu and Roberts 2009; Lee and Łatuszyński 2014). The cause for this is well-known to be related with the value of the ratio between $W_{y,N}$ and $W_{x,N}$ at a particular iteration. Heuristically, when the value of the current weight (*w* in (3)) is large, proposed moves can have a low probability of acceptance. As a consequence, the resulting chain can get “stuck” and may not move after a considerable number of iterations.

In order to overcome this issue, a subtly different algorithm is performed in some practical problems (see, e.g., McKinley et al. 2014). The basic idea is to refresh, independently from the past, the value of the current weight at every iteration. The ratio of the weights between $W_{y,N}$ and $W_{x,N}$ still plays an important role in this alternative algorithm, but here refreshing $W_{x,N}$ at every iteration can improve mixing and the rate of convergence.

This alternative algorithm is commonly known as Monte Carlo within Metropolis (MCWM), as in O’Neill et al. (2000), Beaumont (2003) or Andrieu and Roberts (2009), since typically the weights are Monte Carlo estimates as in (4). From this point onwards it will be referred as the noisy MH algorithm or simply the noisy algorithm to emphasize that our main assumption is (2). Due to independence from previous iterations while sampling $W_{x,N}$ and $W_{y,N}$, the noisy algorithm also defines a time-homogeneous Markov chain with transition kernel ${\tilde{P}}_{N}$, but on the measurable space $(\mathcal {X},\mathcal {B}(\mathcal {X}))$. A single draw from ${\tilde{P}}_{N}(x,\cdot )$ is presented in Algorithm 2, and it is clear that we restrict our attention to strictly positive weights because the algorithm is not well-defined when both $W_{y,N}$ and $W_{x,N}$ are equal to 0.
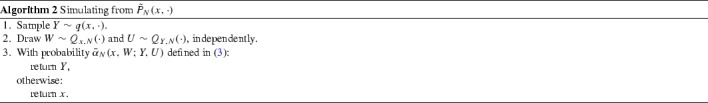


Even though these algorithms differ only slightly, the related chains have very different properties. In Algorithm 2, the value *w* is generated at every iteration whereas in Algorithm 1, it is treated as an input. As a consequence, Algorithm 1 produces a chain on $\left( \mathcal {X}\times \mathcal {W},\mathcal {B}(\mathcal {X})\times \mathcal {B}(\mathcal {W})\right)$ contrasting with a chain from Algorithm 2 taking values on $\left( \mathcal {X},\mathcal {B}(\mathcal {X})\right)$. However, the noisy chain is not invariant under $\pi$ and it is not reversible in general. Moreover, it may not even have an invariant distribution as shown by some examples in Sect. 2.

From O’Neill et al. (2000) and Fernández-Villaverde and Rubio-Ramírez (2007), it is evident that the implementation of the noisy algorithm goes back even before the appearance of the pseudo-marginal, the latter initially conceptualised as Grouped Independence Metropolis–Hastings (GIMH) in Beaumont (2003). Theoretical properties, however, of the noisy algorithm have mainly been studied in tandem with the pseudo-marginal by Beaumont (2003), Andrieu and Roberts (2009) and more recently by Alquier et al. (2014).

The noisy chain generated by Algorithm 2 can be seen as a perturbed version of an idealised Markov chain where the weights $\left\{ W_{x,N}\right\} _{x,N}$ are all equal to one. Perturbed Markov chains have been investigated in , e.g., Roberts et al. (1998), Breyer et al. (2001), Shardlow and Stuart (2000), Mitrophanov (2005), Ferré et al. (2013). More recently Pillai and Smith (2014) and Rudolf and Schweizer (2015) study such chains using the notion of Wasserstein distance. We focus on total variation distance, a particular case of the Wasserstein distance. The relationship between our work and these latter papers is pointed out in subsequent remarks.

**Fig. 2:**
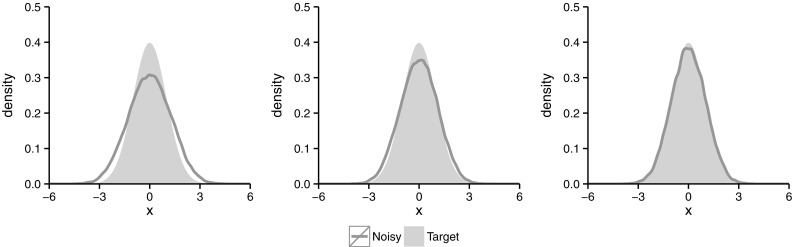
Estimated densities using the noisy chain with 100, 000 iterations for $N=10$ (*left*), $N=100$ (*central*) and $N=1000$ (*right*)

### Objectives of the article

The objectives of this article can be illustrated using a simple example. Let $\mathcal {N}(\cdot | \mu , \sigma ^2)$ denote a univariate Gaussian distribution with mean $\mu$ and variance $\sigma ^2$ and $\pi (\cdot )=\mathcal {N}(\cdot | 0, 1)$ be a standard normal distribution. Let the weights $W_{x,N}$ be as in (4) with

$$\begin{aligned} Q_x(\cdot )=\log \mathcal {N}\left( \cdot \Big |-\frac{1}{2}\sigma ^2, \sigma ^2 \right) \quad \text {and} \quad \sigma ^2:=5, \end{aligned}$$

where $\log \mathcal {N}(\cdot | \mu , \sigma ^2)$ denotes a log-normal distribution of parameters $\mu$ and $\sigma ^2$. In addition, let the proposal *q* be random walk given by $q(x,\cdot )=\mathcal {N}\left( \cdot |x, 4 \right)$. For this example, Fig. 2 shows the estimated densities using the noisy chain for different values of *N*. It appears that the noisy chain has an invariant distribution, and as *N* increases it seems to approach the desired target $\pi$. Our objectives here are to answer the following types of questions about the noisy algorithm in general:

1. Does an invariant distribution exist, at least for *N* large enough?
2. Does the noisy Markov chain behave like the marginal chain for sufficiently large *N*?
3. Does the invariant distribution, if it exists, converge to $\pi$ as *N* increases?

We will see that the answer to the first two questions is negative in general. However, all three questions can be answered positively when the marginal chain is geometrically ergodic and the distributions of the weights satisfy additional assumptions.

### Marginal chains and geometric ergodicity

In order to formalise our analysis, let *P* denote the Markov transition kernel of a standard MH chain on $\left( \mathcal {X},\mathcal {B}(\mathcal {X})\right)$, targeting $\pi$ with proposal *q*. We will refer to this chain and this algorithm using the term marginal (as in Andrieu and Roberts 2009; Andrieu and Vihola 2015), which is the idealised version for which the noisy chain and corresponding algorithm are simple approximations. Therefore

$$\begin{aligned} P(x,dy) := \alpha (x,y)q(x,dy)+\delta _{x}(dy)\rho (x), \end{aligned}$$

where $\alpha$ is the MH acceptance probability and $\rho$ is the rejection probability, given by

5 $$\begin{aligned} \alpha (x,y)&:=\min \left\{ 1,\frac{\pi (dy)q(y,dx)}{\pi (dx)q(x,dy)}\right\} \quad \text {and}\nonumber \\ \rho (x)&:=1-\int _{\mathcal {X}}\alpha (x,y)q(x,dy). \end{aligned}$$

Similarly, for the transition kernel ${\tilde{P}}_{N}$ of the noisy chain, moves are proposed according to *q* but are accepted using ${\bar{\alpha }}_{N}$ (as in (3)) instead of $\alpha$, once values for $W_{x,N}$ and $W_{y,N}$ are sampled. In order to distinguish the acceptance probabilities between the noisy and the pseudo-marginal processes, despite being the same after sampling values for the weights, define

6 $$\begin{aligned} \tilde{\alpha }_{N}(x,y):= {\mathbb {E}}_{Q_{x,N}\otimes Q_{y,N}}{\bar{\alpha }}_{N}(x,W_{x,N};y,W_{y,N}). \end{aligned}$$

Here $\tilde{\alpha }_{N}$ is the expectation of a randomised acceptance probability, which permits defining the transition kernel of the noisy chain by

$$\begin{aligned} {\tilde{P}}_{N}(x,dy) := \tilde{\alpha }_{N}(x,y)q(x,dy)+\delta _{x}(dy)\tilde{\rho }_{N}(x), \end{aligned}$$

where $\tilde{\rho }_{N}$ is the noisy rejection probability given by

7 $$\begin{aligned} \tilde{\rho }_{N}(x) := 1-\int _{\mathcal {X}}\tilde{\alpha }_{N}(x,y)q(x,dy). \end{aligned}$$

As briefly noted before, the noisy kernel ${\tilde{P}}_{N}$ is just a perturbed version of *P* involving a ratio of weights in the noisy acceptance probability $\tilde{\alpha }_{N}$. When such weights are identically one, i.e. $Q_{x,N}(\{1\})=1$, the noisy chain reduces to the marginal chain, whereas the pseudo-marginal becomes the marginal chain with an extra component always equal to 1.

So far, the terms slow convergence and “sticky” behaviour have been used in a relative vague sense. A powerful characterisation of the behaviour of a Markov chain is provided by geometric ergodicity, defined below. Geometrically ergodic Markov chains have a limiting invariant probability distribution, which they converge towards geometrically fast in total variation (Meyn and Tweedie 2009). For any Markov kernel $K:\mathcal {X}\times \mathcal {B}(\mathcal {X})\rightarrow [0,1]$, let $K^{n}$ be the *n*-step transition kernel, which is given by

$$\begin{aligned} K^{n} (x,\cdot ):=\int _{\mathcal {X}}K^{n-1}(x,dz)K(z,\cdot ),\quad \text {for}\;n\ge 2. \end{aligned}$$

#### **Definition 1.1**

(*Geometric ergodicity*) A $\varphi$-irreducible and aperiodic Markov chain $\varvec{\Phi }:=(\varPhi _i)_{i\ge 0}$ on a measurable space $\left( \mathcal {X},\mathcal {B}(\mathcal {X})\right)$, with transition kernel *P* and invariant distribution $\pi$, is geometrically ergodic if there exists a finite function $V\ge 1$ and constants $\tau <1$, $R<\infty$ such that

8 $$\begin{aligned} \Vert P^{n}(x,\cdot )-\pi (\cdot )\Vert _{TV} \le RV(x)\tau ^{n},\quad \text {for}\;x\in \mathcal {X}. \end{aligned}$$

Here, $\Vert \cdot \Vert _{TV}$ denotes the total variation norm given by

$$\begin{aligned} \Vert \mu \Vert _{TV}&= \frac{1}{2} \sup _{|g|\le 1}\Big |\int \mu (dy)g(y)\Big |=\sup _{A\in \mathcal {B}(\mathcal {X})}\mu (A), \end{aligned}$$

where $\mu$ is any signed measure.

Geometric ergodicity does not necessarily provide fast convergence in an absolute sense. For instance, consider cases where $\tau$, or *R*, from Definition 1.1 are extremely close to one, or very large respectively. Then the decay of the total variation distance, though geometric, is not particularly fast (see Roberts and Rosenthal 2004 for some examples).

Nevertheless, geometric ergodicity is a useful tool when analysing non-reversible Markov chains as will become apparent in the noisy chain case. Moreover, in practice one is often interested in estimating ${\mathbb {E}}_{\pi }\left[ f(X)\right]$ for some function $f:\mathcal {X}\rightarrow {\mathbb {R}}$, which is done by using ergodic averages of the form

$$\begin{aligned} e_{n,m}(f) := \frac{1}{n}\sum _{i=m+1}^{m+n} f\left( \varPhi _{i}\right) , \quad \text {for}\;m,n\ge 0. \end{aligned}$$

In this case, geometric ergodicity is a desirable property since it can guarantee the existence of a central limit theorem (CLT) for $e_{n,m}(f)$, see Chan and Geyer (1994) and Roberts and Rosenthal (1997, 2004) for a more general review. Also, its importance is related with the construction of consistent estimators of the corresponding asymptotic variance in the CLT, as in Flegal and Jones (2010).

As noted in Andrieu and Roberts (2009), if the weights $\left\{ W_{x,N}\right\} _{x,N}$ are not essentially bounded then the pseudo-marginal chain cannot be geometrically ergodic; in such cases the “stickiness” may be more evident. In addition, under mild assumptions (in particular, that $\bar{P}_N$ has a left spectral gap), from Andrieu and Vihola (2015, Proposition 10) and Lee and Łatuszyński (2014), a sufficient but not necessary condition ensuring the pseudo-marginal inherits geometric ergodicity from the marginal, is that the weights are uniformly bounded. This certainly imposes a tight restriction in many practical problems.

The analyses in Andrieu and Roberts (2009) and Alquier et al. (2014) mainly study the noisy algorithm in the case where the marginal Markov chain is uniformly ergodic, i.e. when it satisfies (8) with $\sup _{x\in \mathcal {X}} V(x)<\infty$. However, there are many Metropolis–Hastings Markov chains for statistical estimation that cannot be uniformly ergodic, e.g. random walk Metropolis chains when $\pi$ is not compactly supported. Our focus is therefore on inheritance of geometric ergodicity by the noisy chain, complementing existing results for the pseudo-marginal chain.

### Outline of the paper

In Sect. 2, some simple examples are presented for which the noisy chain is positive recurrent, so it has an invariant probability distribution. This is perhaps the weakest stability property that one would expect a Monte Carlo Markov chain to have. However, other fairly surprising examples are presented for which the noisy Markov chain is transient even though the marginal and pseudo-marginal chains are geometrically ergodic. Section 3 is dedicated to inheritance of geometric ergodicity from the marginal chain, where two different sets of sufficient conditions are given and are further analysed in the context of arithmetic averages given by (4). Once geometric ergodicity is attained, it guarantees the existence of an invariant distribution $\tilde{\pi }_{N}$ for the noisy chain. Under the same sets of conditions, we show in Sect. 4 that $\tilde{\pi }_{N}$ and $\pi$ can be made arbitrarily close in total variation as *N* increases. Moreover, explicit rates of convergence are possible to obtain in principle, when the weights arise from an arithmetic average setting as in (4).

## Motivating examples

### Homogeneous weights with a random walk proposal

Assume a log-concave target distribution $\pi$ on the positive integers, whose density with respect to the counting measure is given by

$$\begin{aligned} \pi (m) \propto \exp \left\{ -h(m)\right\} \mathbbm {1}_{m\in {\mathbb {N}}^{+}}, \end{aligned}$$

where $h:{\mathbb {N}}^{+}\rightarrow {\mathbb {R}}$ is a convex function. In addition, let the proposal distribution be a symmetric random walk on the integers, i.e.

9 $$\begin{aligned} q(m,\{m+1\})= \frac{1}{2}=q(m,\{m-1\}),\quad \text {for}\;m\in \mathbb {Z}. \end{aligned}$$

From Mengersen and Tweedie (1996), it can be seen that the marginal chain is geometrically ergodic.

Now, assume the distribution of the weights $\left\{ W_{m,N}\right\} _{m,N}$ is homogeneous with respect to the state space, meaning

10 $$\begin{aligned} W_{m,N}=W_{N} \sim Q_{N}(\cdot ),\quad \text {for all}\;m\in {\mathbb {N}}^{+}. \end{aligned}$$

In addition, assume $W_{N}>0\ Q_{N}$-a.s., then for $m\ge 2$

$$\begin{aligned}&{\tilde{P}}_{N} (m,\left\{ m-1\right\} )\nonumber \\&\quad = \frac{1}{2}{\mathbb {E}}_{Q_{N}\otimes Q_{N}}\left[ \min \left\{ 1,\frac{\exp \{h(m)\}}{\exp \{h(m-1)\}}\times \frac{W_{N}^{(1)}}{W_{N}^{(2)}}\right\} \right] \nonumber \\&\text {and}\\&{\tilde{P}}_{N}(m,\left\{ m+1\right\} )\nonumber \\&\quad = \frac{1}{2}{\mathbb {E}}_{Q_{N}\otimes Q_{N}}\left[ \min \left\{ 1,\frac{\exp \{h(m)\}}{\exp \{h(m+1)\}}\times \frac{W_{N}^{(1)}}{W_{N}^{(2)}}\right\} \right] ,\nonumber \\&\text {where}\;\left\{ W_{N}^{(k)}\right\} _{k\in \{1,2\}}\overset{i.i.d.}{\sim }Q_N(\cdot ). \end{aligned}$$

For this particular class of weights and using the fact that *h* is convex, the noisy chain is geometrically ergodic, implying the existence of an invariant probability distribution.

#### **Proposition 2.1**

Consider a log-concave target density on the positive integers and a proposal density as in (9). In addition, let the distribution of the weights be homogeneous as in (10). Then, the chain generated by the noisy kernel ${\tilde{P}}_{N}$ is geometrically ergodic.

It is worth noting that the distribution of the weights, though homogeneous with respect to the state space, can be taken arbitrarily, as long as the weights are positive. Homogeneity ensures that the distribution of the ratio of such weights is not concentrated near 0, due to its symmetry around one, i.e. for $z>0$

$$\begin{aligned}&{\mathbb {P}}_{Q_{N}\otimes Q_{N}}\left[ \frac{W_{N}^{(1)}}{W_{N}^{(2)}} \le z\right] = {\mathbb {P}}_{Q_{N}\otimes Q_{N}}\left[ \frac{W_{N}^{(1)}}{W_{N}^{(2)}}\ge \frac{1}{z}\right] ,\nonumber \\&\text {where}\; \left\{ W_{N}^{(k)}\right\} _{k\in \{1,2\}}\overset{i.i.d.}{\sim }Q_{N}(\cdot ). \end{aligned}$$

In contrast, when the support of the distribution $Q_{N}$ is unbounded, the corresponding pseudo-marginal chain cannot be geometrically ergodic.

### Particle MCMC

More complex examples arise when using particle MCMC methods, for which noisy versions can also be performed. They may prove to be useful in some inference problems. Consider again the hidden Markov model given by Fig. 1. As before, set $X_{0}=x_{0}$ and let

$$\begin{aligned}&\theta =\left\{ x_{0},a,\sigma _{X}^{2},\sigma _{Y}^{2}\right\} ,\\&f_{\theta }\left( \cdot |X_{t-1}\right) =\mathcal {N}\left( \cdot |aX_{t-1},\sigma _{X}^{2}\right) \quad \text {and}\\&g_{\theta }\left( \cdot |X_{t}\right) = \mathcal {N}\left( \cdot |X_{t},\sigma _{Y}^{2}\right) . \end{aligned}$$

Therefore, once a prior distribution for $\theta$ is specified, $p(\cdot )$ say, the aim is to conduct Bayesian inference on the posterior distribution

$$\begin{aligned} \pi (\theta |y_1,\ldots ,y_T)\propto p(\theta ) l(\theta ;y_1,\ldots ,y_T). \end{aligned}$$

**Fig. 3:**
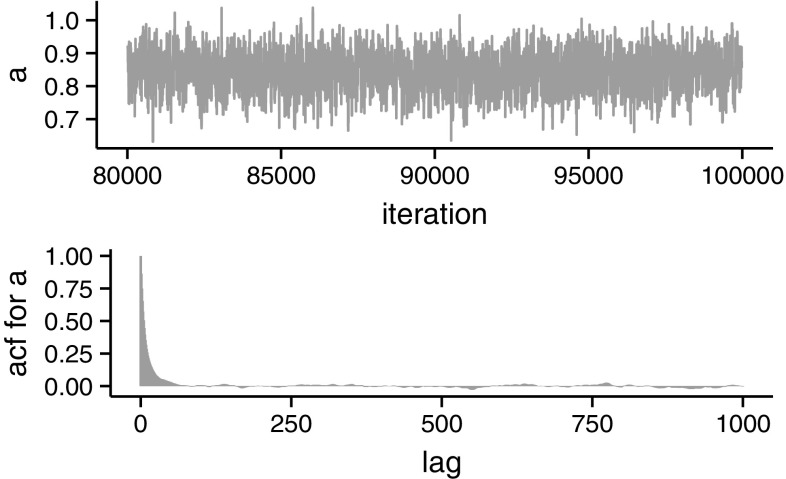
Last 20,000 iterations of the marginal algorithm for the autoregressive parameter *a* (*top*). Estimated autocorrelation function of the corresponding marginal chain (*bottom*). The mean acceptance probability was 0.256

**Fig. 4:**
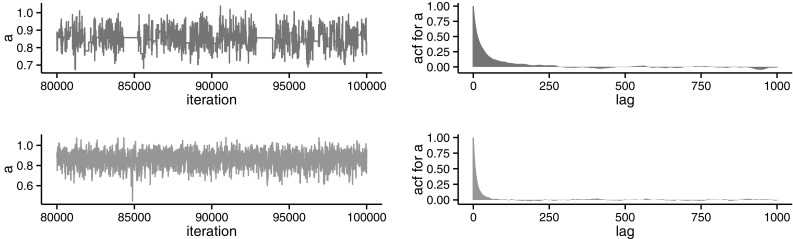
Last 20,000 iterations of the pseudo-marginal (*top left*) and noisy (*bottom left*) algorithms, for the autoregressive parameter *a* when $N=250$. Estimated autocorrelation functions of the corresponding pseudo-marginal (*top right*) and noisy (*bottom right*) chains. The mean acceptance probabilities were 0.104 for the pseudo-marginal and 0.283 for the noisy chain

In this particular setting, the posterior distribution is tractable. This will allows us to compare the results obtained from the exact and noisy versions, both relying on the SMC estimator $\hat{l}_N(\theta ;y_1,\ldots ,y_T)$ of the likelihood. Using a uniform prior for the parameters and a random walk proposal, Fig. 3 shows the run and autocorrelation function (acf) for the autoregressive parameter *a* of the marginal chain. Similarly, Fig. 4 shows the corresponding run and acf for both the pseudo-marginal and the noisy chain when $N=250$. Plots for the other parameters and different values of *N* can be found in Online Appendix 2. It is noticeable how the pseudo-marginal gets “stuck”, resulting in a lower acceptance than the marginal and noisy chains. In addition, the acf of the noisy chain seems to decay faster than that of the pseudo-marginal chain.

**Fig. 5:**
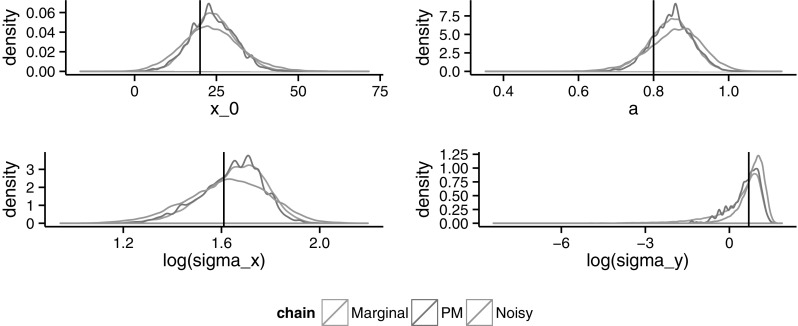
Estimated densities using the marginal, pseudo-marginal and noisy chains for the four parameters when $N=250$. *Vertical lines* indicate the real values

**Fig. 6:**
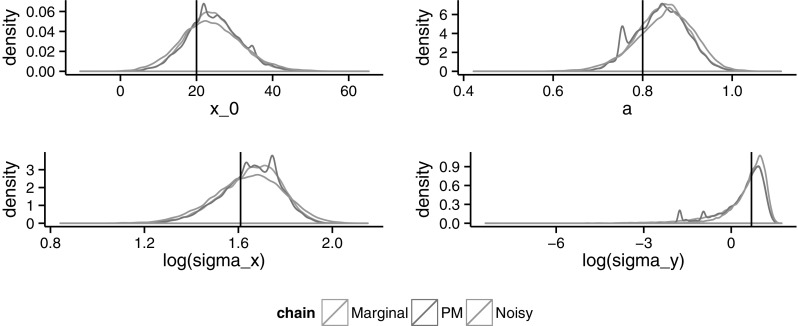
Estimated densities using the marginal, pseudo-marginal and noisy chains for the four parameters, when $N=750$. *Vertical lines* indicate the real values

Finally, Figs. 5 and 6 show the estimated posterior densities for the parameters when $N=250$ and $N=750$, respectively. There, the trade-off between the pseudo-marginal and the noisy algorithm is noticeable. For lower values of *N*, the pseudo-marginal will require more iterations due to the slow mixing, whereas the noisy converges faster towards an unknown noisy invariant distribution. By increasing *N*, the mixing in the pseudo-marginal improves and the noisy invariant approaches the true posterior. Plots for other values of *N* can also be found in Online Appendix 2.

### Transient noisy chain with homogeneous weights

In contrast with example in Sect. 2.1, this one shows that the noisy algorithm can produce a transient chain even in simple settings. Let $\pi$ be a geometric distribution on the positive integers, whose density with respect to the counting measure is given by

11 $$\begin{aligned} \pi (m) = \left( \frac{1}{2}\right) ^{m}\mathbbm {1}_{\left\{ m\in {\mathbb {N}}^{+}\right\} }. \end{aligned}$$

In addition, assume the proposal distribution is a simple random walk on the integers, i.e.

12 $$\begin{aligned} q(m,\{m+1\}) = \theta =1-q(m,\{m-1\}),\quad \text {for}\;m\in \mathbb {Z}. \end{aligned}$$

where $\theta \in (0,1)$. Under these assumptions, the marginal chain is geometrically ergodic, see Proposition 5.1 in Appendix 1.

Consider $N=1$ and as in Sect. 2.1, let the distribution of weights be homogeneous and given by

13 $$\begin{aligned} W = (b-\varepsilon )Ber(s)+\varepsilon ,\quad \text {for}\;b>1\;\text {and}\;\varepsilon \in (0,1), \end{aligned}$$

where *Ber*(*s*) denotes a Bernoulli random variable of parameter $s\in (0,1).$ There exists a relationship between *s*, *b* and $\varepsilon$ that guarantees the expectation of the weights is identically one. The following proposition, proven in Appendix 1 by taking $\theta > 1/2$, shows that the resulting noisy chain can be transient for certain values of *b*, $\epsilon$ and $\theta$.

#### **Proposition 2.2**

Consider a geometric target density as in (11) and a proposal density as in (12). In addition, let the weights when $N=1$ be given by (13). Then, for some *b*, $\varepsilon$ and $\theta$ the chain generated by the noisy kernel ${\tilde{P}}_{N=1}$ is transient.

**Fig. 7:**
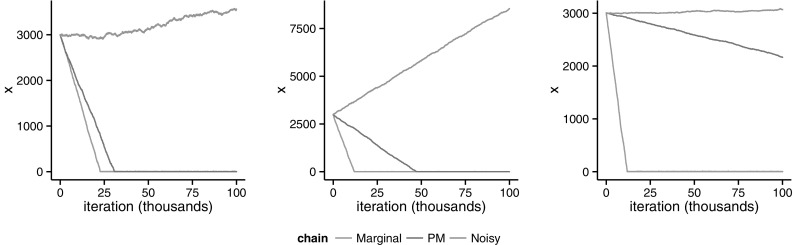
Runs of the marginal, pseudo-marginal and noisy chains. *Left plot* shows example in Sect. 2.3, where $\theta =0.75$, $\varepsilon =2-\sqrt{3}$ and $b=2\varepsilon \frac{\theta }{1-\theta }$. *Central* and *right plots* show example in Sect. 2.4, where $\theta =0.5$ and $\theta =0.25$ respectively, with $\varepsilon _{m}=m^{-(3-m\ (\text {mod}\ 3))}$ and $b=3+\left( \frac{1-\theta }{\theta }\right) ^{3}$

In contrast, since the weights are uniformly bounded by *b*, the pseudo-marginal chain inherits geometric ergodicity for any $\theta$, *b* and $\epsilon$. The left plot in Fig. 7 shows an example. We will discuss the behaviour of this example as *N* increases in Sect. 3.4 .

### Transient noisy chain with non-homogeneous weights

One could argue that the transient behaviour of the previous example is related to the large value of $\theta$ in the proposal distribution. However, as shown here, for any value of $\theta \in (0,1)$ one can construct weights satisfying (2) for which the noisy chain is transient. With the same assumptions as in the example in Sect. 2.3, except that now the distribution of weights is not homogeneous but given by

14 $$\begin{aligned}&W_{m,1} = (b-\varepsilon _{m})Ber(s_{m})+\varepsilon _{m},\nonumber \\&\text {for}\;b>1\;\text {and}\;\varepsilon _{m}=m^{-(3-(m\ (\text {mod}\ 3)))}, \end{aligned}$$

the noisy chain will be transient for *b* large enough. The proof can be found in Appendix 1.

#### **Proposition 2.3**

Consider a geometric target density as in (11) and a proposal density as in (12). In addition, let the weights when $N=1$ be given by (14). Then, for any $\theta \in (0,1)$ there exists some $b>1$ such that the chain generated by the noisy kernel ${\tilde{P}}_{N=1}$ is transient.

The reason for this becomes apparent when looking at the behaviour of the ratios of weights. Even though $\varepsilon _{m}\rightarrow 0$ as $m\rightarrow \infty$, the non-monotonic behaviour of the sequence implies

$$\begin{aligned} \frac{\varepsilon _{m-1}}{\varepsilon _{m}}\in&{\left\{ \begin{array}{ll} \begin{array}{l} O\left( m^{2}\right) \\ O\left( m^{-1}\right) \end{array}\quad \text {if} &{} \begin{array}{l} m\ (\text {mod}\ 3)=0,\\ m\ (\text {mod}\ 3)\in \{1,2\}, \end{array}\end{array}\right. } \end{aligned}$$

and

$$\begin{aligned} \frac{\varepsilon _{m+1}}{\varepsilon _{m}}\in&{\left\{ \begin{array}{ll} \begin{array}{l} O\left( m^{-2}\right) \\ O\left( m\right) \end{array}\quad \text {if} &{} \begin{array}{l} m\ (\text {mod}\ 3)=2,\\ m\ (\text {mod}\ 3)\in \{0,1\}. \end{array}\end{array}\right. } \end{aligned}$$

Hence, the ratio of the weights can become arbitrarily large or arbitrarily close to zero with a non-negligible probability. This allows the algorithm to accept moves to the right more often, if *m* is large enough. Once again, the pseudo-marginal chain inherits the geometrically ergodic property from the marginal. See the central and right plots of Fig. 7 for two examples using different proposals. Again, we will come back to this example in Sect. 3.4, where we look at the behaviour of the associated noisy chain as *N* increases.

## Inheritance of ergodic properties

The inheritance of various ergodic properties of the marginal chain by pseudo-marginal Markov chains has been established using techniques that are powerful but suitable only for reversible Markov chains (see, e.g. Andrieu and Vihola 2015). Since the noisy Markov chains treated here can be non-reversible, a suitable tool for establishing geometric ergodicity is the use of Foster–Lyapunov functions, via geometric drift towards a small set.

### **Definition 3.1**

(*Small set*) Let *P* be the transition kernel of a Markov chain $\varvec{\Phi }$. A subset $C\subseteq \mathcal {X}$ is small if there exists a positive integer $n_{0}$, $\varepsilon >0$ and a probability measure $\nu (\cdot )$ on $\left( \mathcal {X},\mathcal {B}(\mathcal {X})\right)$ such that the following minorisation condition holds

15 $$\begin{aligned} P^{n_{0}}(x,\cdot )&\ge \varepsilon \nu (\cdot ),\qquad \text {for}\;x\in C. \end{aligned}$$

The following theorem, which is immediate from combining Roberts and Rosenthal (1997, Proposition 2.1) and Meyn and Tweedie (2009, Theorem 15.0.1), establishes the equivalence between geometric ergodicity and a geometric drift condition. For any kernel $K:\mathcal {X}\times \mathcal {B}(\mathcal {X})\rightarrow [0,1]$, let

$$\begin{aligned} KV (x):=\int _{\mathcal {X}}K(x,dz)V(z). \end{aligned}$$

### **Theorem 3.1**

Suppose that $\varPhi$ is a $\phi$-irreducible and aperiodic Markov chain with transition kernel *P* and invariant distribution $\pi$. Then, the following statements are equivalent:

(i) There exists a small set *C*, constants $\lambda <1$ and $b<\infty$, and a function $V\ge 1$ finite for some $x_0\in \mathcal {X}$ satisfying the geometric drift condition
  16 $$\begin{aligned} PV(x) \le \lambda V(x)+b\mathbbm {1}_{\left\{ x\in C\right\} },\quad \text {for}\;x\in \mathcal {X}. \end{aligned}$$
(ii) The chain is $\pi$-a.e. geometrically ergodic, meaning that for $\pi$-a.e. $x\in \mathcal {X}$ it satisfies (8) for some $V\ge 1$ (which can be taken as in (i)) and constants $\tau <1$, $R<\infty$.

From this point onwards, it is assumed that the marginal and noisy chains are $\phi$-irreducible and aperiodic. In addition, for many of the following results, it is required that

(**P1**): The marginal chain is geometrically ergodic, implying its kernel *P* satisfies the geometric drift condition in (16) for some constants $\lambda <1$ and $b<\infty$, some function $V\ge 1$ and a small set $C\subseteq \mathcal {X}$.

### Conditions involving a negative moment

From the examples of the previous section, it is clear that the weights play a fundamental role in the behaviour of the noisy chain. The following theorem states that the noisy chain will inherit geometric ergodicity from the marginal under some conditions on the weights involving a strengthened version of the Law of Large Numbers and convergence of negative moments.

(**W1**): For any $\delta >0$, the weights $\left\{ W_{x,N}\right\} _{x,N}$ satisfy
  $$\begin{aligned} \lim _{N\rightarrow \infty }\sup _{x\in \mathcal {X}}{\mathbb {P}}_{Q_{x,N}}\left[ \big \vert W_{x,N}-1\big \vert \ge \delta \right] = 0. \end{aligned}$$
(**W2**): The weights $\left\{ W_{x,N}\right\} _{x,N}$ satisfy
  $$\begin{aligned} \lim _{N\rightarrow \infty }\sup _{x\in \mathcal {X}}{\mathbb {E}}_{Q_{x,N}}\left[ W_{x,N}^{-1}\right] = 1. \end{aligned}$$

#### **Theorem 3.2**

Assume (P1), (W1) and (W2). Then, there exists $N_{0}\in {\mathbb {N}}^{+}$ such that for all $N\ge N_{0}$, the noisy chain with transition kernel ${\tilde{P}}_{N}$ is geometrically ergodic.

The above result is obtained by controlling the dissimilarity of the marginal and noisy kernels. This is done by looking at the corresponding rejection and acceptance probabilities. The proofs of the following lemmas appear in Appendix 1.

#### **Lemma 3.1**

For any $$\delta >0$$

$$\begin{aligned}&{\mathbb {P}}_{Q_{z,N} \otimes Q_{x,N}}\left[ \frac{W_{z,N}}{W_{x,N}}\le 1-\delta \right] \nonumber \\&\le 2\sup _{x\in \mathcal {X}}{\mathbb {P}}_{Q_{x,N}}\left[ \Big \vert W_{x,N}-1\Big \vert \ge \frac{\delta }{2}\right] . \end{aligned}$$

#### **Lemma 3.2**

Let $\rho (x)$ and $\tilde{\rho }_{N}(x)$ be the rejection probabilities as defined in (5) and (7) respectively. Then, for any $\delta >0$

$$\begin{aligned} \tilde{\rho }_{N}(x)-\rho (x)\le \delta +2\sup _{x\in \mathcal {X}}{\mathbb {P}}_{Q_{x,N}}\left[ \big \vert W_{x,N}-1\big \vert \ge \frac{\delta }{2}\right] . \end{aligned}$$

#### **Lemma 3.3**

Let $\alpha (x,y)$ and $\tilde{\alpha }_{N}(x,y)$ be the acceptance probabilities as defined in (5) and (6) respectively. Then,

$$\begin{aligned} \tilde{\alpha }_{N}(x,y) \le \alpha (x,y){\mathbb {E}}_{Q_{x,N}}\left[ W_{x,N}^{-1}\right] . \end{aligned}$$

Notice that (W1) and (W2) allow control on the bounds in the above lemmas. While Lemma 3.2 provides a bound for the difference of the rejection probabilities, Lemma 3.3 gives one for the ratio of the acceptance probabilities. The proof of Theorem 3.2 is now presented.

#### *Proof of Theorem 3.2*

Since the marginal chain *P* is geometrically ergodic, it satisfies the geometric drift condition in (16) for some $\lambda <1$, $b<\infty$, some function $V\ge 1$ and a small set $C\subseteq \mathcal {X}$. Now, using the above lemmas

$$\begin{aligned}&{\tilde{P}}_{N}V(x) -PV(x)\nonumber \\&\quad = \int _{\mathcal {X}}q(x,dz)\left( \tilde{\alpha }_{N}(x,z)-\alpha (x,z)\right) V(z)\nonumber \\&\qquad +V(x)\left( \tilde{\rho }_{N}(x)-\rho (x)\right) \nonumber \\&\quad \le \left( \sup _{x\in \mathcal {X}}{\mathbb {E}}\left[ W_{x,N}^{-1}\right] -1\right) PV(x)\nonumber \\&\qquad +\left( \delta +2\sup _{x\in \mathcal {X}}{\mathbb {P}}\left[ \big \vert W_{x,N}-1\big \vert \ge \frac{\delta }{2}\right] \right) V(x). \end{aligned}$$

By (W1) and (W2), for any $\varepsilon$, $\delta >0$ there exists $N_{0}\in {\mathbb {N}}^{+}$ such that

$$\begin{aligned}&\sup _{x\in \mathcal {X}}{\mathbb {P}}\left[ \big \vert W_{x,N}-1\big \vert \ge \frac{\delta }{2}\right] <\frac{\varepsilon }{4} \quad \text {and}\\&\sup _{x\in \mathcal {X}}{\mathbb {E}}\left[ W_{x,N}^{-1}\right] -1<\varepsilon , \end{aligned}$$

whenever $$N\ge N_{0}$$, implying

$$\begin{aligned} {\tilde{P}}_{N}V(x)&\le PV(x)+\varepsilon PV(x)+\left( \delta +\frac{\varepsilon }{2}\right) V(x)\nonumber \\&\le \left( \lambda + \varepsilon \lambda +\delta +\frac{\varepsilon }{2}\right) V(x)+b\left( 1+\varepsilon \right) \mathbbm {1}_{\left\{ x\in C\right\} }. \end{aligned}$$

Taking $\delta =\frac{\varepsilon }{2}$ and $\varepsilon \in \left( 0,\frac{1-\lambda }{1+\lambda }\right)$, the noisy chain ${\tilde{P}}_{N}$ also satisfies a geometric drift condition for the same function *V* and small set *C*, completing the proof. $\square$

#### *Remark 3.1*

In fact, (W1) and (W2) together guarantee for any $\delta >0$

$$\begin{aligned} \delta \le \tilde{\alpha }_N(x,y) - \alpha (x,y)\le \alpha (x,y) \delta , \end{aligned}$$

which is the crucial assumption in Pillai and Smith (2014, Lemma 3.6) for obtaining a similar drift condition.

### Conditions on the proposal distribution

In this subsection a different bound for the acceptance probabilities is provided, which allows dropping assumption (W2) but imposes a different one on the proposal *q* instead.

$$(\mathbf{P1}^{*})$$
(P1) holds and for the same drift function *V* in (P1) there exists $K<\infty$ such that the proposal kernel *q* satisfies
  $$\begin{aligned} qV(x) \le KV(x),\quad \text {for}\;x\in \mathcal {X}. \end{aligned}$$

#### **Theorem 3.3**

Assume (P1*) and (W1). Then, there exists $N_{0}\in {\mathbb {N}}^{+}$ such that for all $N\ge N_{0}$, the noisy chain with transition kernel ${\tilde{P}}_{N}$ is geometrically ergodic.

In order to prove Theorem 3.3 the following lemma is required. Its proof can be found in Appendix 1. In contrast with Lemma 3.3, this lemma provides a bound for the additive difference of the noisy and marginal acceptance probabilities.

#### **Lemma 3.4**

Let $\alpha (x,y)$ and $\tilde{\alpha }_{N}(x,y)$ be the acceptance probabilities as defined in (5) and (6), respectively. Then, for any $\eta >0$

$$\begin{aligned}&\tilde{\alpha }_{N}(x,y) -\alpha (x,y)\nonumber \\&\quad \le \eta +2\sup _{x\in \mathcal {X}}{\mathbb {P}}_{Q_{x,N}}\left[ \Big \vert W_{x,N}-1\Big \vert \ge \frac{\eta }{2\left( 1+\eta \right) }\right] . \end{aligned}$$

#### *Proof of Theorem 3.3*

Using Lemmas 3.2 and 3.4 with $\eta =\delta$

$$\begin{aligned}&{\tilde{P}}_{N}V(x)-PV(x)\nonumber \\&\quad = \int _{\mathcal {X}}q(x,dz)\left( \tilde{\alpha }_{N}(x,z)-\alpha (x,z)\right) V(z)\nonumber \\&\qquad +V(x)\left( \tilde{\rho }_{N}(x)-\rho (x)\right) \nonumber \\&\quad \le \left( \delta +2\sup _{x\in \mathcal {X}}{\mathbb {P}}\left[ \Big \vert W_{x,N}-1\Big \vert \ge \frac{\delta }{2\left( 1+\delta \right) }\right] \right) qV(x)\nonumber \\&\qquad +\left( \delta +2\sup _{x\in \mathcal {X}}{\mathbb {P}}\left[ \Big \vert W_{x,N}-1\Big \vert \ge \frac{\delta }{2}\right] \right) V(x)\nonumber \\&\quad \le \left( \delta +2\sup _{x\in \mathcal {X}}{\mathbb {P}}\left[ \Big \vert W_{x,N}-1\Big \vert \ge \frac{\delta }{2\left( 1+\delta \right) }\right] \right) \nonumber \\&\qquad \times \left( qV(x)+V(x)\right) . \end{aligned}$$

By (W1), there exists $N_{0}\in {\mathbb {N}}^{+}$ such that

$$\begin{aligned} \sup _{x\in \mathcal {X}}{\mathbb {P}}\left[ \Big \vert W_{x,N}-1\Big \vert \ge \frac{\delta }{2\left( 1+\delta \right) }\right]&<\frac{\varepsilon }{4}, \end{aligned}$$

whenever $N\ge N_{0}$. This implies

$$\begin{aligned} {\tilde{P}}_{N}V(x) \le PV(x)+\left( \delta +\frac{\varepsilon }{2}\right) \left( qV(x)+V(x)\right) , \end{aligned}$$

and using (P1*)

$$\begin{aligned} {\tilde{P}}_{N}V(x) \le \left( \lambda +\left( \delta +\frac{\varepsilon }{2}\right) \left( K+1\right) \right) V(x)+b\mathbbm {1}_{\left\{ x\in C\right\} }. \end{aligned}$$

Taking $\delta =\frac{\varepsilon }{2}$ and $\varepsilon \in \left( 0,\frac{1-\lambda }{1+K}\right)$, the noisy chain ${\tilde{P}}_{N}$ also satisfies a geometric drift condition for the same function *V* and small set *C*, completing the proof. $\square$

#### *Remark 3.2*

By itself, (W1) implies for any $\delta >0$

$$\begin{aligned} |\tilde{\alpha }_N(x,y) - \alpha (x,y)| \le \delta , \end{aligned}$$

but it needs to be paired with (P1*) to obtain the desired result. These assumptions are comparable to those in Pillai and Smith (2014, Lemma 3.6), taking *f* constant therein. Additionally, (W1) and (P1*) imply the required conditions on $\mathcal {E}$ and $\lambda$ in Rudolf and Schweizer (2015, Corollary 31), where a similar result is proved in terms of *V*-uniform ergodicity.

In general, assumption (P1*) may be difficult to verify as one must identify a particular function *V*, but it is easily satisfied when restricting to log-Lipschitz targets and when using a random walk proposal of the form

17 $$\begin{aligned} q(x,dy) = q(\Vert y-x\Vert )dy, \end{aligned}$$

where $\Vert \cdot \Vert$ denotes the usual Euclidean distance. To see this the following assumption is required, which is a particular case of (P1) and is satisfied under some extra technical conditions (see, e.g., Roberts and Tweedie 1996).

$$(\mathbf{P1}^{**})$$
$\mathcal {X}\subseteq {\mathbb {R}}^{d}$. The target $\pi$ is log-Lipschitz, meaning that for some $L>0$
  $$\begin{aligned} |\log \pi (z)-\log \pi (x)| \le L\Vert z-x\Vert . \end{aligned}$$
  (P1) holds taking the drift function $V=\pi ^{-s}$, for any $s\in (0,1)$. The proposal *q* is a random walk as in (17) satisfying
  $$\begin{aligned} \int _{{\mathbb {R}}^{d}}\exp \left\{ a\Vert u\Vert \right\} q(\Vert u\Vert )du < \infty , \end{aligned}$$
  for some $$a>0$$.

See Appendix 1 for a proof of the following proposition.

#### **Proposition 3.1**

Assume $$(\mathrm{P}1^{**})$$ and (W1). Then, (P1*) holds.

### Conditions for arithmetic averages

In the particular setting where the weights are given by (4), sufficient conditions on these can be obtained to ensure geometric ergodicity is inherited by the noisy chain. For the simple case where the weights are homogeneous with respect to the state space (W1) is automatically satisfied. In order to attain (W2), the existence of a negative moment for a single weight is required. See Appendix 1 for a proof of the following result.

#### **Proposition 3.2**

Assume weights as in (4). If  ${\mathbb {E}}_{Q_x}\left[ W_x^{-1}\right] <\infty$ then

18 $$\begin{aligned} \lim _{N\rightarrow \infty }{\mathbb {E}}_{Q_{x,N}}\left[ W_{x,N}^{-1}\right] = 1. \end{aligned}$$

For homogeneous weights, (18) implies (W2). When the weights are not homogeneous, stronger conditions are needed for (W1) and (W2) to be satisfied. An appropriate first assumption is that the weights are uniformly integrable.

(**W3**): The weights $\left\{ W_{x}\right\} _{x}$ satisfy
  $$\begin{aligned} \lim _{K\rightarrow \infty }\sup _{x\in \mathcal {X}}{\mathbb {E}}_{Q_{x}}\left[ W_{x}\mathbbm {1}_{\{W_{x}>K\}}\right] =0. \end{aligned}$$

The second condition imposes an additional assumption on the distribution of the weights $\left\{ W_{x}\right\} _{x}$ near 0.

(**W4**): There exists $\gamma \in (0,1)$ and constants $M<\infty$, $\beta >0$ such that for $w\in (0,\gamma )$ the weights $\left\{ W_{x}\right\} _{x}$ satisfy
  $$\begin{aligned} \sup _{x\in \mathcal {X}} {\mathbb {P}}_{Q_x} \left[ W_x \le w \right] \le M w^{\beta }. \end{aligned}$$

These new conditions ensure (W1) and (W2) are satisfied.

#### **Proposition 3.3**

For weights as in (4),

(i) (W3) implies (W1);
(ii) (W1) and (W4) imply (W2).

The following corollary is obtained as an immediate consequence of the above proposition, Theorems 3.2 and 3.3.

#### **Corollary 3.1**

Let the weights be as in (4). Assume (W3) and either

(i) (P1) and (W4);
(ii) (P1*).

Then, there exists $N_{0}\in {\mathbb {N}}^{+}$ such that for all $N\ge N_{0}$, the noisy chain with transition kernel ${\tilde{P}}_{N}$ is geometrically ergodic.

The proof of Proposition 3.3 follows the statement of Lemma 3.5, whose proof can be found in Appendix 1. This lemma allows us to characterise the distribution of $W_{x,N}$ near 0 assuming (W4) and also provides conditions for the existence and convergence of negative moments.

#### **Lemma 3.5**

Let $$\gamma \in (0,1)$$ and $$p>0$$.

(i) Suppose *Z* is a positive random variable, and assume that for $z\in (0,\gamma )$
  $$\begin{aligned} {\mathbb {P}}\left[ Z \le z \right] \le Mz^{\alpha },\quad \text {where}\;\alpha >p,M<\infty . \end{aligned}$$
  Then,
  $$\begin{aligned} {\mathbb {E}}\left[ Z^{-p} \right] \le \frac{1}{\gamma ^p} + pM\frac{\gamma ^{\alpha -p}}{\alpha -p}. \end{aligned}$$
(ii) Suppose $\left\{ Z_{i}\right\} _{i=1}^{N}$ is a collection of positive and independent random variables, and assume that for each $i\in \left\{ 1,\ldots ,N\right\}$ and $z\in (0,\gamma )$
  $$\begin{aligned} {\mathbb {P}}\left[ Z_{i} \le z \right] \le M_{i} z^{\alpha _{i}},\quad \text {where}\;\alpha _{i}>0,M_{i}<\infty . \end{aligned}$$
  Then, for $$z\in (0,\gamma )$$
  $$\begin{aligned} {\mathbb {P}}\left[ \sum _{i=1}^{N}Z_{i}\le z\right] \le \prod _{i=1}^N M_i z^{\sum _{i=1}^{N}\alpha _{i}}. \end{aligned}$$
(iii) Let the weights be as in (4). If for some $N_0\in {\mathbb {N}}^+$
  $$\begin{aligned} {\mathbb {E}}_{Q_{x,N_0}}\left[ W_{x,N_0}^{-p} \right] < \infty , \end{aligned}$$
  then for any $N\ge N_0$
  $$\begin{aligned} {\mathbb {E}}_{Q_{x,N+1}}\left[ W_{x,N+1}^{-p} \right] \le {\mathbb {E}}_{Q_{x,N}}\left[ W_{x,N}^{-p} \right] . \end{aligned}$$
(iv) Assume (W1) and let $g:{\mathbb {R}}^{+}\rightarrow {\mathbb {R}}$ be a function that is continuous at 1 and bounded on the interval $[\gamma ,\infty )$. Then
  $$\begin{aligned} \lim _{N\rightarrow \infty }\sup _{x\in \mathcal {X}}{\mathbb {E}}_{Q_{x,N}}\left[ |g\left( W_{x,N}\right) -g\left( 1\right) |\mathbbm {1}_{W_{x,N}\ge \gamma }\right] = 0. \end{aligned}$$

#### *Proof of Proposition 3.3*

Part (i) is a consequence of Chandra (1989, Theorem 1). Assuming (W3), it implies

$$\begin{aligned} \lim _{N\rightarrow \infty }\sup _{x\in \mathcal {X}}{\mathbb {E}}\big \vert W_{x,N}-1\big \vert = 0. \end{aligned}$$

By Markov’s inequality

$$\begin{aligned} {\mathbb {E}}\big \vert W_{x,N}-1\big \vert \ge \delta {\mathbb {P}}\left[ \big \vert W_{x,N}-1\big \vert \ge \delta \right] , \end{aligned}$$

and the result follows.

To prove (ii), assume (W4) and by part (ii) of Lemma 3.5, for $w\in (0,\gamma )$

$$\begin{aligned} {\mathbb {P}}\left[ N W_{x,N} \le w \right] \le M^N w^{N\beta }. \end{aligned}$$

Take $p>1$ and define $N_0:=\lfloor \frac{p}{\beta } \rfloor +1$, then using part (i) of Lemma 3.5 if $N\ge N_0$

$$\begin{aligned} \sup _{x\in \mathcal {X}}{\mathbb {E}}\left[ W_{x,N}^{-p} \right] \le \frac{N}{\gamma ^p} + pNM^N \frac{\gamma ^{N\beta -p}}{N\beta -p}. \end{aligned}$$

Hence, by Hölder’s inequality

$$\begin{aligned}&{\mathbb {E}} \left[ \big \vert W_{x,N}^{-1}-1\big \vert \mathbbm {1}_{W_{x,N}\in (0,\gamma )}\right] \nonumber \\&\quad \le {\mathbb {E}}\left[ W_{x,N}^{-1} \mathbbm {1}_{W_{x,N}\in (0,\gamma )}\right] \nonumber \\&\quad \le \left( {\mathbb {E}}\left[ W_{x,N}^{-p} \right] \right) ^\frac{1}{p} \left( {\mathbb {P}}\left[ W_{x,N} < \gamma \right] \right) ^{\frac{p-1}{p}}, \end{aligned}$$

and applying part (iii) of Lemma 3.5, for $N\ge N_0$

$$\begin{aligned}&{\mathbb {E}} \left[ \big \vert W_{x,N}^{-1}-1\big \vert \mathbbm {1}_{W_{x,N}\in (0,\gamma )}\right] \nonumber \\&\quad \le \left( {\mathbb {E}}\left[ W_{x,N_0}^{-p} \right] \right) ^\frac{1}{p} \left( {\mathbb {P}}\left[ W_{x,N} < \gamma \right] \right) ^{\frac{p-1}{p}}. \end{aligned}$$

Therefore,

$$\begin{aligned}&\sup _{x\in \mathcal {X}}{\mathbb {E}} \left[ \big \vert W_{x,N}^{-1}-1\big \vert \mathbbm {1}_{W_{x,N}\in (0,\gamma )}\right] \nonumber \\&\quad \le \left( \sup _{x\in \mathcal {X}} {\mathbb {E}}\left[ W_{x,N_0}^{-p} \right] \right) ^\frac{1}{p} \left( \sup _{x\in \mathcal {X}} {\mathbb {P}}\left[ W_{x,N} < \gamma \right] \right) ^{\frac{p-1}{p}}. \end{aligned}$$

Since $\gamma <1$ and by (W1)

$$\begin{aligned} \lim _{N\rightarrow \infty }\sup _{x\in \mathcal {X}} {\mathbb {P}}\left[ W_{x,N} < \gamma \right] =0, \end{aligned}$$

implying

19 $$\begin{aligned} \lim _{N\rightarrow \infty }\sup _{x\in \mathcal {X}}{\mathbb {E}} \left[ \big \vert W_{x,N}^{-1}-1\big \vert \mathbbm {1}_{W_{x,N}\in (0,\gamma )}\right] =0. \end{aligned}$$

Now, for fixed $\gamma \in (0,1)$ the function $g(x)=x^{-1}$ is bounded and continuous on $[\gamma ,\infty )$, implying by part (iv) of Lemma 3.5

20 $$\begin{aligned} \lim _{N\rightarrow \infty }\sup _{x\in \mathcal {X}}{\mathbb {E}}\left[ \big \vert W_{x,N}^{-1}-1\big \vert \mathbbm {1}_{W_{x,N}\in [\gamma ,\infty )}\right] = 0. \end{aligned}$$

Finally, using (19) and (20)

$$\begin{aligned} \lim _{N\rightarrow \infty }\sup _{x\in \mathcal {X}} {\mathbb {E}}\big \vert W_{x,N}^{-1}-1\big \vert = 0, \end{aligned}$$

and by the triangle inequality

$$\begin{aligned} \sup _{x\in \mathcal {X}}{\mathbb {E}}\big \vert W_{x,N}^{-1}-1\big \vert \ge \sup _{x\in \mathcal {X}}{\mathbb {E}}\left[ W_{x,N}^{-1}\right] -1, \end{aligned}$$

the result follows. $\square$

### Remarks on results

Equipped with these results, we return to the examples in Sects. 2.3 and 2.4. Even though the noisy chain can be transient in these examples, the behaviour is quite different when considering weights that are arithmetic averages of the form in (4). Since in both examples the weights are uniformly bounded by the constant *b*, they immediately satisfy (W1). Additionally, by Proposition 3.2, condition (W2) is satisfied for the example in Sect. 2.3. This is not the case for example in Sect. 2.4, but condition (P1*) is satisfied by taking $V=\pi ^{-\frac{1}{2}}$. Therefore, applying Theorems 3.2 and 3.3 to examples in Sects. 2.3 and 2.4 respectively, as *N* increases the corresponding chains will go from being transient to geometrically ergodic.

Despite conditions (W1) and (W2) guaranteeing the inheritance of geometric ergodicity for the noisy chain, they are not necessary. Consider a modification of the example in Sect. 2.3, where the weights are given by

$$\begin{aligned}&W_{m,1} = (b_{m}-\varepsilon _{m})Ber(s_{m})+\varepsilon _{m},\nonumber \\&\text {where}\;b_{m}>1\;\text {and}\;\varepsilon _{m}\in (0,1]\;\text {for all}\;m\ge 1. \end{aligned}$$

Again, there exists a relationship between the variables $b_{m}$, $\varepsilon _{m}$ and $s_{m}$ for ensuring the expectation of the weights is equal to one. Let $Bin\left( N,s\right)$ denote a binomial distribution of parameters $N\in {\mathbb {N}}^{+}$ and $s\in (0,1)$. Then, in the arithmetic average context, $W_{m,N}$ becomes

21 $$\begin{aligned}&W_{m,N} = \frac{\left( b_{m}-\varepsilon _{m}\right) }{N}Bin\left( N,s_{m}\right) +\varepsilon _{m},\nonumber \\&\text {where}\;b_{m}>1\;\text {and}\;\varepsilon _{m}\in (0,1]\;\text {for all}\;m\ge 1. \end{aligned}$$

For particular choices of the sequences $\left\{ b_{m}\right\} _{m\in {\mathbb {N}}^{+}}$ and $\left\{ \varepsilon _{m}\right\} _{m\in {\mathbb {N}}^{+}}$, the resulting noisy chain can be geometrically ergodic for all $N\ge 1$, even though neither (W1) nor (W2) hold.

#### **Proposition 3.4**

Consider a geometric target density as in (11) and a proposal density as in (12). In addition, let the weights be as in (21) with $b_{m}\rightarrow \infty$, $\varepsilon _{m}\rightarrow 0$ as $m\rightarrow \infty$ and

$$\begin{aligned} \lim _{m\rightarrow \infty }\frac{\varepsilon _{m-1}}{\varepsilon _{m}} = l,\quad \text {where}\;l\in {\mathbb {R}}^{+}\cup \left\{ +\infty \right\} . \end{aligned}$$

Then, the chain generated by the noisy kernel ${\tilde{P}}_{N}$ is geometrically ergodic for any $N\in {\mathbb {N}}^{+}$.

Finally, in many of the previous examples, increasing the value of *N* seems to improve the ergodic properties of the noisy chain. However, the geometric ergodicity property is not always inherited, no matter how large *N* is taken. The following proposition shows an example rather similar to Proposition 3.4, but in which the ratio $\frac{\varepsilon _{m-1}}{\varepsilon _{m}}$ does not converge as $m\rightarrow \infty$.

#### **Proposition 3.5**

Consider a geometric target density as in (11) and a proposal density as in (12). In addition, let the weights be as in (21) with $b_{m}=m$ and

$$\begin{aligned} \varepsilon _{m} = m^{-(3-(m\ (\text {mod}\ 3)))}. \end{aligned}$$

Then, the chain generated by the noisy kernel ${\tilde{P}}_{N}$ is transient for any $N\in {\mathbb {N}}^{+}$.

## Convergence of the noisy invariant distribution

So far the only concern has been whether the noisy chain inherits the geometric ergodicity property from the marginal chain. As an immediate consequence, geometric ergodicity guarantees the existence of an invariant probability distribution $\tilde{\pi }_{N}$ for ${\tilde{P}}_{N}$, provided *N* is large enough. In addition, using the same conditions from Sect. 3, we can characterise and in some cases quantify the convergence in total variation of $\tilde{\pi }_{N}$ towards the desired target $\pi$, as $N\rightarrow \infty$.

### Convergence in total variation

The following definition, taken from Roberts et al. (1998), characterises a class of kernels satisfying a geometric drift condition as in (16) for the same *V*, *C*, $\lambda$ and *b*.

#### **Definition 4.1**

(*Simultaneous geometric ergodicity*) A class of Markov chain kernels $\left\{ P_{k}\right\} _{k\in \mathcal {K}}$ is simultaneously geometrically ergodic if there exists a class of probability measures $\left\{ \nu _{k}\right\} _{k\in \mathcal {K}}$, a measurable set $C\subseteq \mathcal {X}$, a real valued measurable function $V\ge 1$, a positive integer $n_{0}$ and positive constants $\varepsilon$, $\lambda$, *b* such that for each $k\in \mathcal {K}$:

(i) *C* is small for $P_{k}$, with $P_{k}^{n_{0}}(x,\cdot )\ge \varepsilon \nu _{k}(\cdot )$ for all $x\in C$;
(ii) the chain $P_{k}$ satisfies the geometric drift condition in (16) with drift function *V*, small set *C* and constants $\lambda$ and *b*.

Provided *N* is large, the noisy kernels $\{ {\tilde{P}}_{N+k}\}_{k\ge 0}$ together with the marginal *P* will be simultaneous geometrically ergodic. This will allow the use of coupling arguments for ensuring $\tilde{\pi }_{N}$ and $\pi$ get arbitrarily close in total variation. The main additional assumption is

(**P2**): For some $\varepsilon >0$, some probability measure $\nu (\cdot )$ on $\left( \mathcal {X},\mathcal {B}(\mathcal {X})\right)$ and some subset $C\subseteq \mathcal {X}$, the marginal acceptance probability $\alpha$ and the proposal kernel *q* satisfy
  $$\begin{aligned} \alpha (x,y)q(x,dy) \ge \varepsilon \nu (dy), \quad \text {for}\;x\in C. \end{aligned}$$

#### *Remark 4.1*

(P2) ensures the marginal chain satisfies the minorisation condition in (15), purely attained by the sub-kernel $\alpha (x,y)q(x,dy)$. This occurs under fairly mild assumptions (see, e.g., Roberts and Tweedie 1996, Theorem 2.2).

#### **Theorem 4.1**

Assume (P1), (P2), (W1) and (W2). Alternatively, assume (P1*), (P2) and (W1). Then,

(i) there exists $N_{0}\in {\mathbb {N}}^{+}$ such that the class of kernels $\left\{ P,{\tilde{P}}_{N_{0}},{\tilde{P}}_{N_{0}+1},\ldots \right\}$ is simultaneously geometrically ergodic;
(ii) for all $x\in \mathcal {X}$, $\lim _{N\rightarrow \infty }\Vert {\tilde{P}}_{N}(x,\cdot )-P(x,\cdot )\Vert _{TV}=0$;
(iii) $$\lim _{N\rightarrow \infty }\Vert \tilde{\pi }_{N}(\cdot )-\pi (\cdot )\Vert _{TV}=0.$$

Part (iii) of the above theorem is mainly a consequence of Roberts et al. (1998, Theorem 9) when parts (i) and (ii) hold. Indeed, by the triangle inequality,

22 $$\begin{aligned}&\Vert \tilde{\pi }_{N}(\cdot ) - \pi (\cdot )\Vert _{TV}\nonumber \\&\quad \le \Vert {\tilde{P}}_{N}^{n}(x,\cdot )-\tilde{\pi }_{N}(\cdot )\Vert _{TV}+\Vert P^{n}(x,\cdot )-\pi (\cdot )\Vert _{TV}\nonumber \\&\qquad +\Vert {\tilde{P}}_{N}^{n}(x,\cdot )-P^{n}(x,\cdot )\Vert _{TV}. \end{aligned}$$

Provided $N\ge N_{0}$, the first two terms in (22) can be made arbitrarily small by increasing *n*. In addition, due to the simultaneous geometrically ergodic property, the first term in (22) is uniformly controlled regardless the value of *N*. Finally, using an inductive argument, part (ii) implies that for all $x\in \mathcal {X}$ and all $n\in {\mathbb {N}}^{+}$

$$\begin{aligned} \lim _{N\rightarrow \infty }\Vert {\tilde{P}}_{N}^{n}(x,\cdot )-P^{n}(x,\cdot )\Vert _{TV} = 0. \end{aligned}$$

#### *Proof of Theorem 4.1*

From the proofs of Theorems 3.2 and 3.3, there exists $N_{1}\in {\mathbb {N}}^{+}$ such that the class of kernels $\left\{ P,{\tilde{P}}_{N_{1}},{\tilde{P}}_{N_{1}+1},\ldots \right\}$ satisfies condition (ii) in Definition 4.1 for the same function *V*, small set *C* and constants $\lambda _{N_{1}},b_{N_{1}}$. Respecting (i), for any $\delta \in (0,1)$

$$\begin{aligned}&{\tilde{P}}_{N} (x,A)\nonumber \\&\quad \ge \int _{A}\tilde{\alpha }_{N}(x,z)q(x,dz)\nonumber \\&\quad \ge \int _{A}{\mathbb {E}}\left[ \min \left\{ 1,\frac{W_{z,N}}{W_{x,N}}\right\} \right] \alpha (x,z)q(x,dz)\nonumber \\&\quad \ge (1-\delta ) \nonumber \\&\qquad \times \int _{A}\left( 1-{\mathbb {P}}\left[ \frac{W_{z,N}}{W_{x,N}}\le 1-\delta \right] \right) \alpha (x,z)q(x,dz). \end{aligned}$$

Then, by Lemma 3.1

$$\begin{aligned}&{\tilde{P}}_{N} (x,A)\nonumber \\&\quad \ge (1-\delta )\left( 1-2\sup _{x\in \mathcal {X}}{\mathbb {P}}\left[ \Big \vert W_{x,N}-1\Big \vert \ge \frac{\delta }{2}\right] \right) \nonumber \\&\qquad \times \int _{A}\alpha (x,z)q(x,dz). \end{aligned}$$

By (W1), there exists $N_{2}\in {\mathbb {N}}^{+}$ such that for $N\ge N_{2}$

$$\begin{aligned} \sup _{x\in \mathcal {X}}{\mathbb {P}}\left[ \Big \vert W_{x,N}-1\Big \vert \ge \frac{\delta }{2}\right] \le \frac{\delta }{2}, \end{aligned}$$

giving

$$\begin{aligned} {\tilde{P}}_{N}(x,A) \ge (1-\delta )^{2}\int _{A}\alpha (x,z)q(x,dz). \end{aligned}$$

Due to (P2),

$$\begin{aligned} {\tilde{P}}_{N}(x,A) \ge (1-\delta )^{2}\varepsilon \nu (A),\quad \text {for}\;x\in C. \end{aligned}$$

Finally, take $N_{0}=\max \left\{ N_{1},N_{2}\right\}$ implying (i).

To prove (ii) apply Lemma 3.2 and Lemma 3.4 to get

23 $$\begin{aligned}&\sup _{A\in \mathcal {B}(\mathcal {X})} \left\{ {\tilde{P}}_{N}(x,A)-P(x,A)\right\} \nonumber \\&\quad \le \left( \eta +2\sup _{x\in \mathcal {X}}{\mathbb {P}}_{Q_{x,N}}\left[ \Big \vert W_{x,N}-1\Big \vert \ge \frac{\eta }{2\left( 1+\eta \right) }\right] \right) \nonumber \\&\qquad \times \sup _{A\in \mathcal {B}(\mathcal {X})}q(x,A) \nonumber \\&\qquad +\left( \tilde{\rho _{N}}(x)-\rho (x)\right) \sup _{A\in \mathcal {B}(\mathcal {X})}\mathbbm {1}_{x\in A} \nonumber \\&\quad \le \left( \eta +2\sup _{x\in \mathcal {X}}{\mathbb {P}}_{Q_{x,N}}\left[ \Big \vert W_{x,N}-1\Big \vert \ge \frac{\eta }{2\left( 1+\eta \right) }\right] \right) \nonumber \\&\qquad +\left( \delta +2\sup _{x\in \mathcal {X}}{\mathbb {P}}_{Q_{x,N}}\left[ \big \vert W_{x,N}-1\big \vert \ge \frac{\delta }{2}\right] \right) \end{aligned}$$

Finally, taking $N\rightarrow \infty$ and by (W1)

$$\begin{aligned} \lim _{N\rightarrow \infty }\sup _{A\in \mathcal {B}(\mathcal {X})}\left\{ {\tilde{P}}_{N}(x,A)-P(x,A)\right\} \le \eta +\delta . \end{aligned}$$

The result follows since $\eta$ and $\delta$ can be taken arbitrarily small.

For (iii), see Theorem 9 in Roberts et al. (1998) for a detailed proof. $\square$

#### *Remark 4.2*

A Wasserstein distance variant of part (iii) in Theorem 4.1 has been proved in Rudolf and Schweizer (2015, Corollary 28), in which control of the difference between $\tilde{\alpha }_N$ and $\alpha$ is still required and can be obtained using (W1).

### Rate of convergence

Let $(\tilde{\varPhi }_{n}^{N})_{n\ge 0}$ denote the noisy chain and $\left( \varPhi _{n}\right) _{n\ge 0}$ the marginal chain, which move according to the kernels ${\tilde{P}}_{N}$ and *P*, respectively and define $c_{x}:=1-\Vert {\tilde{P}}_{N}(x,\cdot )-P(x,\cdot )\Vert _{TV}$. Using notions of maximal coupling for random variables defined on a Polish space (see Lindvall 2002 and Thorisson 2013), there exists a probability measure $\nu _{x}(\cdot )$ such that

$$\begin{aligned} P(x,\cdot )\ge c_{x}\nu _{x}(\cdot )\quad \text {and}\quad {\tilde{P}}_{N}(x,\cdot )\ge c_{x}\nu _{x}(\cdot ). \end{aligned}$$

Let $c:=\inf _{x\in \mathcal {X}}c_{x}$, define a coupling in the following way

- If $\tilde{\varPhi }_{n-1}^{N}=\varPhi _{n-1}=y$, with probability *c* draw $\varPhi _{n}\sim \nu _{y}(\cdot )$ and set $\tilde{\varPhi }_{n}^{N}=\varPhi _{n}$. Otherwise, draw independently $\varPhi _{n}\sim R(y,\cdot )$ and $\tilde{\varPhi }_{n}^{N}\sim \tilde{R}_{N}(y,\cdot )$, where
  $$\begin{aligned} R(y,\cdot )&:= \left( 1-c\right) ^{-1}\left( P(y,\cdot )-c\nu _{y}(\cdot )\right) \quad \text {and}\nonumber \\ \tilde{R}_{N}(y,\cdot )&:= \left( 1-c\right) ^{-1}\left( {\tilde{P}}_{N}(y,\cdot )-c\nu _{y}(\cdot )\right) . \end{aligned}$$
- If $\tilde{\varPhi }_{n-1}^{N}\ne \varPhi _{n-1}$, draw independently $\varPhi _{n}\sim P(y,\cdot )$ and $\tilde{\varPhi }_{n}^{N}\sim {\tilde{P}}_{N}(y,\cdot )$.

Since

$$\begin{aligned}&{\mathbb {P}}\left[ \tilde{\varPhi }_{n}^{N} \ne \varPhi _{n}|\tilde{\varPhi }_{0}^{N}=\varPhi _{0}=x\right] \nonumber \\&\quad \le {\mathbb {P}}\left[ \tilde{\varPhi }_{n}^{N}\ne \varPhi _{n}|\tilde{\varPhi }_{n-1}^{N}=\varPhi _{n-1},\tilde{\varPhi }_{0}^{N}=\varPhi _{0}=x\right] \nonumber \\&\qquad +{\mathbb {P}}\left[ \tilde{\varPhi }_{n-1}^{N}\ne \varPhi _{n-1}|\tilde{\varPhi }_{0}^{N}=\varPhi _{0}=x\right] \nonumber \\&\quad \le 1-c+{\mathbb {P}}\left[ \tilde{\varPhi }_{n-1}^{N}\ne \varPhi _{n-1}|\tilde{\varPhi }_{0}^{N}=\varPhi _{0}=x\right] , \end{aligned}$$

and noting

$$\begin{aligned}&{\mathbb {P}}\left[ \tilde{\varPhi }_{1}^{N}\ne \varPhi _{1}|\tilde{\varPhi }_{0}^{N}=\varPhi _{0}=x\right] \nonumber \\&\quad \le \sup _{x\in \mathcal {X}}\Vert {\tilde{P}}_{N}(x,\cdot )-P(x,\cdot )\Vert _{TV}\nonumber \\&\quad = 1-c, \end{aligned}$$

an induction argument can be applied to obtain

$$\begin{aligned}&{\mathbb {P}}\Big [ \tilde{\varPhi }_{n}^{N}\ne \varPhi _{n} |\tilde{\varPhi }_{0}^{N}=\varPhi _{0}=x\Big ]\nonumber \\&\quad \le n\sup _{x\in \mathcal {X}}\Vert {\tilde{P}}_{N}(x,\cdot )-P(x,\cdot )\Vert _{TV}. \end{aligned}$$

Therefore, using the coupling inequality, the third term in (22) can be bounded by

24 $$\begin{aligned}&\Vert {\tilde{P}}_{N}^{n}(x,\cdot ) -P^{n}(x,\cdot )\Vert _{TV}\nonumber \\&\quad \le {\mathbb {P}}\left[ \tilde{\varPhi }_{n}^{N}\ne \varPhi _{n}|\tilde{\varPhi }_{0}^{N}=\varPhi _{0}=x\right] \nonumber \\&\quad \le n\sup _{x\in \mathcal {X}}\Vert {\tilde{P}}_{N}(x,\cdot )-P(x,\cdot )\Vert _{TV}. \end{aligned}$$

On the other hand, using the simultaneous geometric ergodicity of the kernels and provided *N* is large enough, the noisy and marginal kernels will each satisfy a geometric drift condition as in (16) with a common drift function $V\ge 1$, small set *C* and constants $\lambda ,b$. Therefore, by Theorem 3.1, there exist $R>0$, and $\tau <1$ such that

25 $$\begin{aligned}&\Vert {\tilde{P}}_{N}^{n}(x,\cdot )-\tilde{\pi }_{N}(\cdot )\Vert _{TV}\le RV(x)\tau ^{n}\quad \text {and}\nonumber \\&\quad \Vert P^{n}(x,\cdot )-\pi (\cdot )\Vert _{TV}\le RV(x)\tau ^{n}. \end{aligned}$$

Explicit values for *R* and $\tau$ are in principle possible, as done in Rosenthal (1995) and Meyn and Tweedie (1994). For simplicity assume $\inf _{x\in \mathcal {X}}V(x)=1$, then combining (24) and (25) in (22), for all $n\in {\mathbb {N}}^{+}$

26 $$\begin{aligned}&\Vert \tilde{\pi }_{N}(\cdot ) -\pi (\cdot )\Vert _{TV}\nonumber \\&\quad \le 2R\tau ^{n}+n\sup _{x\in \mathcal {X}}\Vert {\tilde{P}}_{N}(x,\cdot )-P(x,\cdot )\Vert _{TV}. \end{aligned}$$

So, if an analytic expression in terms of *N* is available for the second term on the right hand side of (26), it will be possible to obtain an explicit rate of convergence for $\tilde{\pi }_N$ and $\pi$.

#### **Theorem 4.2**

Assume (P1), (P2), (W1) and (W2). Alternatively, assume (P1*), (P2) and (W1). In addition, suppose

$$\begin{aligned} \sup _{x\in \mathcal {X}}\Vert {\tilde{P}}_{N}(x,\cdot )-P(x,\cdot )\Vert _{TV} \le \frac{1}{r(N)}, \end{aligned}$$

where $r:{\mathbb {N}}^{+}\rightarrow {\mathbb {R}}^{+}$ and $\lim _{N\rightarrow \infty }r(N)=+\infty$. Then, there exists $D>0$ and $N_{0}\in {\mathbb {N}}^{+}$ such that for all $N\ge N_{0}$,

27 $$\begin{aligned} \Vert \tilde{\pi }_{N}(\cdot )-\pi (\cdot )\Vert _{TV} \le D\frac{\log \left( r(N)\right) }{r(N)}. \end{aligned}$$

#### *Proof*

Let $R>0,\tau \in (0,1)$ and $r>0$. Pick *r* large enough, such that

$$\begin{aligned} \log \left( 2Rr\log \left( \tau ^{-1}\right) \right) \ge 1, \end{aligned}$$

then the convex function $f:[1,\infty )\rightarrow {\mathbb {R}}^{+}$ where

$$\begin{aligned} f(s) = 2R\tau ^{s}+\frac{s}{r}, \end{aligned}$$

is minimised at

$$\begin{aligned} s_{*} = \frac{\log \left( 2Rr\log \left( \tau ^{-1}\right) \right) }{\log \left( \tau ^{-1}\right) }. \end{aligned}$$

Restricting the domain of *f* to the positive integers and due to convexity, it is then minimised at either

$$\begin{aligned} n_{1} = \lfloor s_{*}\rfloor \qquad \text {or}\qquad n_{2}=\lceil s_{*}\rceil . \end{aligned}$$

In any case

$$\begin{aligned}&\min \left\{ f(n_{1}),f(n_{2})\right\} \nonumber \\&\quad \le f(s_{*}+1),\nonumber \\&\quad = \frac{1}{r}\left( 1+\frac{\tau +\log \left( 2Rr\log \left( \tau ^{-1}\right) \right) }{\log \left( \tau ^{-1}\right) }\right) . \end{aligned}$$

Finally take *N* large enough such that

$$\begin{aligned} \log \left( 2Rr(N)\log \left( \tau ^{-1}\right) \right) \ge&1, \end{aligned}$$

and from (26)

$$\begin{aligned}&\Vert \tilde{\pi }_{N}(\cdot ) -\pi (\cdot )\Vert _{TV}\nonumber \\&\quad \le \min \left\{ f(n_{1}),f(n_{2})\right\} \nonumber \\&\quad \le \frac{1}{r(N)}\left( 1+\frac{\tau +\log \left( 2Rr(N)\log \left( \tau ^{-1}\right) \right) }{\log \left( \tau ^{-1}\right) }\right) \nonumber \\&\quad = O\left( \frac{\log \left( r(N)\right) }{r(N)}\right) , \end{aligned}$$

obtaining the result. $\square$

#### *Remark 4.3*

A general result bounding the total variation between the law of a Markov chain and a perturbed version is presented in Rudolf and Schweizer (2015, Theorem 21). This is done using the connection between the *V*-norm distance and the Wasserstein distance introduced in Hairer and Mattingly (2011). With such a result, and considering the same assumptions in Theorem 4.2, one could in principle obtain an explicit value for *D* in (27).

Moreover, when the weights are expressed in terms of arithmetic averages as in (4), an explicit expression for *r*(*N*) can be obtained whenever there exists a uniformly bounded moment. This is a slightly stronger assumption than (W3).

(**W5**): There exists $k>0$, such that the weights $\left\{ W_{x}\right\} _{x}$ satisfy
  $$\begin{aligned} \sup _{x\in \mathcal {X}}{\mathbb {E}}_{Q_{x}}\left[ W_{x}^{1+k}\right] < \infty . \end{aligned}$$

#### **Proposition 4.1**

Assume (P1), (P2), (W4) and (W5). Alternatively, assume (P1*), (P2) and (W5). Then, there exists $D_{k}>0$ and $N_{0}\in {\mathbb {N}}^{+}$ such that for all $N\ge N_{0}$,

$$\begin{aligned} \Vert \tilde{\pi }_{N}(\cdot )-\pi (\cdot )\Vert _{TV} \le D_{k}\frac{\log \left( N\right) }{N^{1-\frac{2}{2+k}}}. \end{aligned}$$

If in addition (W5) holds for all $k>0$, then for any $\varepsilon \in (0,1)$ there will exist $D_{\varepsilon }>0$ and $N_{0}\in {\mathbb {N}}^{+}$ such that for all $N\ge N_{0}$,

$$\begin{aligned} \Vert \tilde{\pi }_{N}(\cdot )-\pi (\cdot )\Vert _{TV} \le D_{\varepsilon }\frac{\log \left( N\right) }{N^{1-\varepsilon }}. \end{aligned}$$

## Discussion

In this article, fundamental stability properties of the noisy algorithm have been explored. The noisy Markov kernels considered are perturbed Metropolis–Hastings kernels defined by a collection of state-dependent distributions for non-negative weights all with expectation 1. The general results do not assume a specific form for these weights, which can be simple arithmetic averages or more complex random variables. The former may arise when unbiased importance sampling estimates of a target density are used, while the latter may arise when such densities are estimated unbiasedly using a particle filter.

Two different sets of sufficient conditions were provided under which the noisy chain inherits geometric ergodicity from the marginal chain. The first pair of conditions, (W1) and (W2), involve a stronger version of the Law of Large Numbers for the weights and uniform convergence of the first negative moment, respectively. For the second set, (W1) is still required but (W2) can be replaced with (P1*), which imposes a condition on the proposal distribution. These conditions also imply simultaneous geometric ergodicity of a sequence of noisy Markov kernels together with the marginal Markov kernel, which then ensures that the noisy invariant $\tilde{\pi }_{N}$ converges to $\pi$ in total variation as *N* increases. Moreover, an explicit bound for the rate of convergence between $\tilde{\pi }_{N}$ and $\pi$ is possible whenever an explicit bound (that is uniform in *x*) is available for the convergence between ${\tilde{P}}_{N}(x,\cdot )$ and $P(x,\cdot )$.

When weights are arithmetic averages as in (4), specific conditions were given for inheriting geometric ergodicity from the corresponding marginal chain. The uniform integrability condition in (W3) ensures that (W1) is satisfied, whereas (W4) is essential for satisfying (W2). Regarding the noisy invariant distribution $\tilde{\pi }_{N}$, (W5), which is slightly stronger than (W3), leads to an explicit bound on the rate of convergence of this distribution to $\pi$.

The noisy algorithm remains undefined when the weights have positive probability of being zero. If both weights were zero one could accept the move, reject the move or keep sampling new weights until one of them is not zero. Each of these lead to different behaviour.

As seen in the examples of Sect. 3.4, the behaviour of the ratio of the weights (at least in the tails of the target) plays an important role in the ergodic properties of the noisy chain. In this context, it seems plausible to obtain geometric noisy chains, even when the marginal is not, if the ratio of the weights decays sufficiently fast to zero in the tails. Another interesting possibility, that may lead to future research, is to relax the condition on the expectation of the weights to be identically one.

### Electronic supplementary material

Below is the link to the electronic supplementary material.

Supplementary material 1 (pdf 1719 KB)

## Appendix 1: Proofs

### On state-dependent random walks

The following proposition for state-dependent Markov chains on the positive integers will be useful for addressing some proofs. See Norris (1999) for a proof of parts (*i*) and (ii), for part (iii) see Callaert and Keilson (1973), which is proved within the birth-death process context.

#### **Proposition 5.1**

Suppose we have a random walk $\varPhi$ on ${\mathbb {N}}^{+}$ with transition kernel *P*. Define for $m\ge 1$

$$\begin{aligned} p_{m} := P(m,\left\{ m+1\right\} )\quad \text {and}\quad q_{m}:=P(m,\left\{ m-1\right\} ), \end{aligned}$$

with $q_{1}=0,p_{1}\in (0,1]$ and $p_{m},q_{m}>0,p_{m}+q_{m}\le 1$ for all $m\ge 2$. The resulting chain is:

(i) recurrent if and only if
  $$\begin{aligned} \sum _{m=2}^{\infty }\prod _{i=2}^{m}\frac{q_{i}}{p_{i}}\rightarrow \infty ; \end{aligned}$$
(ii) positive recurrent if and only if
  $$\begin{aligned} \sum _{m=2}^{\infty }\prod _{i=2}^{m}\frac{p_{i-1}}{q_{i}} < \infty ; \end{aligned}$$
(iii) geometrically ergodic if
  $$\begin{aligned} \lim _{m\rightarrow \infty }p_{m} < \lim _{m\rightarrow \infty }q_{m}. \end{aligned}$$

#### *Remark 5.1*

Notice that (iii) is not an if and only if statement and that it implies (ii). Additionally, if the chain is not state-dependent, (ii) implies (iii).

### Section 2

#### *Proof of Proposition 2.1*

Since *h* is convex

$$\begin{aligned} h(m)-h(m-1)&\ge h'(m-1) \quad \text {and}\\ h(m)-h(m+1)&\le -h'(m), \end{aligned}$$

implying

$$\begin{aligned}&\frac{{\tilde{P}}_{N}(m,\left\{ m-1\right\} )}{{\tilde{P}}_{N} (m,\left\{ m+1\right\} )}\nonumber \\&\quad \ge \frac{{\mathbb {E}}\left[ \min \left\{ 1,\exp \{h'(m-1)\}\frac{W_{N}^{(1)}}{W_{N}^{(2)}}\right\} \right] }{{\mathbb {E}}\left[ \min \left\{ 1,\exp \{-h'(m)\}\frac{W_{N}^{(1)}}{W_{N}^{(2)}}\right\} \right] }. \end{aligned}$$

Define $Z:=\frac{W_{N}^{(1)}}{W_{N}^{(2)}}$, and since $\pi (m)\rightarrow 0$ it is true that

28 $$\begin{aligned} \log (k):=\lim _{m\rightarrow \infty }h'(m) > 0, \end{aligned}$$

hence

29 $$\begin{aligned} \lim _{m\rightarrow \infty }\frac{{\tilde{P}}_{N}(m,\left\{ m-1\right\} )}{{\tilde{P}}_{N}(m,\left\{ m+1\right\} )} \ge \frac{{\mathbb {E}}\left[ \min \left\{ 1,kZ\right\} \right] }{{\mathbb {E}}\left[ \min \left\{ 1,k^{-1}Z\right\} \right] }. \end{aligned}$$

If $k=+\infty$, it is clear that the limit in (29) diverges, consequently the noisy chain is geometrically ergodic according to Proposition 5.1. If $k<\infty$, the noisy chain will be geometrically ergodic if

$$\begin{aligned} {\mathbb {E}}\left[ \min \left\{ 1,kZ\right\} \right] > {\mathbb {E}}\left[ \min \left\{ 1,k^{-1}Z\right\} \right] , \end{aligned}$$

which can be translated to

$$\begin{aligned}&k{\mathbb {E}}\left[ Z\mathbbm {1}_{\{Z\le k^{-1}\}}\right] + {\mathbb {P}}\left[ Z>k^{-1}\right] \nonumber \\&\quad >k^{-1}{\mathbb {E}}\left[ Z\mathbbm {1}_{\{Z<k\}}\right] +{\mathbb {P}}\left[ Z\ge k\right] , \end{aligned}$$

or equivalently to

30 $$\begin{aligned}&k{\mathbb {P}}\left[ k^{-1}<Z<k\right] +\left( k^{2}-1\right) {\mathbb {E}}\left[ Z\mathbbm {1}_{\left\{ Z\le k^{-1}\right\} }\right] \nonumber \\&\quad > {\mathbb {E}}\left[ Z\mathbbm {1}_{\left\{ k^{-1}<Z<k\right\} }\right] . \end{aligned}$$

Now consider two cases, first if ${\mathbb {P}}\left[ k^{-1}<Z<k\right] >0$ then it is clear that

$$\begin{aligned} {\mathbb {E}}\left[ \left( k-Z\right) \mathbbm {1}_{\left\{ k^{-1}< Z<k\right\} }\right] > 0, \end{aligned}$$

which satisfies (30). Finally, if ${\mathbb {P}}\left[ k^{-1}<Z<k\right] =0$ then

$$\begin{aligned} {\mathbb {P}}\left[ Z\le k^{-1}\right] = \frac{1}{2}={ \mathbb {P}}\left[ Z\ge k\right] , \end{aligned}$$

implying from (28)

$$\begin{aligned} \left( k^{2}-1\right) {\mathbb {E}}\left[ Z\mathbbm {1}_{\left\{ Z\le k^{-1}\right\} }\right] > 0, \end{aligned}$$

and leading to (30). $\square$

#### *Proof of Proposition 2.2*

For simplicity the subscript *N* is dropped. In this case,

$$\begin{aligned} Q_{m,1} =Q=\mathcal {L}\left( (b-\varepsilon )Ber(s)+\varepsilon \right) , \end{aligned}$$

and the condition ${\mathbb {E}}_{Q}\left[ W\right] =1$ implies

31 $$\begin{aligned} s= \frac{1-\varepsilon }{b-\varepsilon }. \end{aligned}$$

Let $\theta \in \left( \frac{1}{1+2\varepsilon },1\right)$ and set

32 $$\begin{aligned} b= \varepsilon \frac{2\theta }{1-\theta }, \end{aligned}$$

this implies ${\bar{\alpha }}(m,w;m-1,u)\equiv 1$ and

$$\begin{aligned} {\bar{\alpha }}(m,w;m+1,u) = {\left\{ \begin{array}{ll} \begin{array}{l} \frac{1-\theta }{2\theta }\\ 1\\ \left( \frac{1-\theta }{2\theta }\right) ^{2} \end{array}\quad \text {if} &{} \begin{array}{l} u=w\\ u=b,w=\varepsilon \\ u=\varepsilon ,w=b \end{array}\end{array}\right. }. \end{aligned}$$

Therefore, for $$m\ge 2$$,

$$\begin{aligned} \tilde{\alpha }(m, m-1)&= 1\quad \text {and}\\ \tilde{\alpha }(m, m+1)&= \frac{1-\theta }{2\theta }\left( s^{2}+\left( 1-s\right) ^{2}\right) \\&\quad +\left( 1+\left( \frac{1-\theta }{2\theta }\right) ^{2}\right) s\left( 1-s\right) . \end{aligned}$$

Consequently, $${\tilde{P}}(m,\left\{ m-1\right\} )=1-\theta $$ and

$$\begin{aligned}&{\tilde{P}}(m, \left\{ m+1\right\} )\nonumber \\&\quad = \theta \left( \frac{1-\theta }{2\theta }\left( s^{2}+ \left( 1-s\right) ^{2}\right) \right. \nonumber \\&\left. \qquad +\left( 1+\left( \frac{1-\theta }{2\theta }\right) ^{2}\right) s\left( 1-s\right) \right) \nonumber \\&\quad > \theta s(1-s). \end{aligned}$$

From Proposition 5.1, if

$$\begin{aligned} {\tilde{P}}(m,\left\{ m+1\right\} ) > {\tilde{P}}(m,\left\{ m-1\right\} ), \end{aligned}$$

then the noisy chain will be transient. For this to happen, it is enough to pick $\theta$ and *s* such that

$$\begin{aligned} \theta s(1-s)-(1-\theta )\ge 0. \end{aligned}$$

Let $s=\varepsilon$, then from (31) and (32)

33 $$\begin{aligned} \theta&= \frac{(1-\varepsilon +\varepsilon ^{2})}{1-\varepsilon +3\varepsilon ^{2}}\nonumber \\&= 1-\frac{2\varepsilon ^{2}}{1-\varepsilon +3\varepsilon ^{2}}, \end{aligned}$$

and if $\varepsilon \le 2-\sqrt{3}$ then

$$\begin{aligned}&\theta s(1-s)-(1-\theta )\nonumber \\&\quad = \frac{\varepsilon }{1-\varepsilon +3\varepsilon ^{2}}\left( (1-\varepsilon +\varepsilon ^{2})(1-\varepsilon )-2\varepsilon \right) \nonumber \\&\quad \ge \frac{\varepsilon }{1-\varepsilon +3\varepsilon ^{2}}\left( (1-\varepsilon )^{2}-2\varepsilon \right) \nonumber \\&\quad = \frac{\varepsilon }{1-\varepsilon +3\varepsilon ^{2}}\left( (2-\varepsilon )^{2}-3\right) \nonumber \\&\quad \ge 0. \end{aligned}$$

Hence, for $\varepsilon \in \left( 0,2-\sqrt{3}\right)$ and setting $s=\varepsilon$, $\theta$ as in (33) and *b* as in (32), the resulting noisy chain is transient. $\square$

#### *Proof of Proposition 2.3*

For simplicity the subscript *N* is dropped. In this case,

$$\begin{aligned} Q_{m,1} = Q_{m}=\mathcal {L}\left( (b-\varepsilon _{m})\textit{Ber}(s_{m})+\varepsilon _{m}\right) , \end{aligned}$$

and the condition ${\mathbb {E}}_{Q_{m}}\left[ W_{m}\right] =1$ implies

$$\begin{aligned} s_{m} = \frac{1-\varepsilon _{m}}{b-\varepsilon _{m}}. \end{aligned}$$

Then, for *m* large enough

$$\begin{aligned} \tilde{\alpha }(m,m-1)&= {\mathbb {E}}\left[ {\bar{\alpha }}(m,W_{m};m-1,W_{m-1})\right] \nonumber \\&= \min \left\{ 1,\frac{2\theta }{1-\theta }\right\} s_{m-1}s_{m}+s_{m-1}(1-s_{m})\nonumber \\&\quad +(1-s_{m-1})(1-s_{m})\mathbbm {1}_{\left\{ m\ (\text {mod}\ 3)=0\right\} }\nonumber \\&\quad +O\left( m^{-1}\right) \quad \text {and}\nonumber \\ \tilde{\alpha }(m,m-1)&= {\mathbb {E}}\left[ {\bar{\alpha }}(m,W_{m};m-1,W_{m-1})\right] \nonumber \\&= \min \left\{ 1,\frac{1-\theta }{2\theta }\right\} s_{m}s_{m+1}+(1-s_{m})s_{m+1}\nonumber \\&\quad +(1-s_{m})(1-s_{m+1})\mathbbm {1}_{\left\{ m\ (\text {mod}\ 3)\ne 2\right\} }\nonumber \\&\quad +O\left( m^{-1}\right) . \end{aligned}$$

Define

$$\begin{aligned} c_{m}&:= \frac{{\tilde{P}}(m,\left\{ m-1\right\} )}{{\tilde{P}}(m,\left\{ m+1\right\} )}\nonumber \\&= \frac{(1-\theta )\tilde{\alpha }(m,m-1)}{\theta \tilde{\alpha }(m,m+1)}. \end{aligned}$$

Since $$s_{m}\rightarrow \frac{1}{b}$$ as $$m\rightarrow \infty $$,

$$\begin{aligned} c_{0,\infty }&:= \lim _{k\rightarrow \infty }c_{3k}\nonumber \\&= \left( \frac{1-\theta }{\theta }\right) \nonumber \\&\quad \times \frac{\left( \min \left\{ 1,\frac{2\theta }{1-\theta }\right\} -1\right) \frac{1}{b^{2}}+\frac{1}{b}+\left( 1-\frac{1}{b}\right) ^{2}}{\left( \min \left\{ 1,\frac{1-\theta }{2\theta }\right\} -1\right) \frac{1}{b^{2}}+\frac{1}{b}+\left( 1-\frac{1}{b}\right) ^{2}}\nonumber \\&\le \left( \frac{1-\theta }{\theta }\right) \frac{1}{1-\frac{1}{b}}\nonumber \\&= \left( \frac{1-\theta }{\theta }\right) \frac{b}{b-1}=:l_{0},\\ c_{1,\infty }:=&\lim _{k\rightarrow \infty }c_{3k}c_{3k+1}\nonumber \\&= c_{0,\infty }\left( \frac{1-\theta }{\theta }\right) \nonumber \\&\quad \times \frac{\left( \min \left\{ 1,\frac{2\theta }{1-\theta }\right\} -1\right) \frac{1}{b^{2}}+\frac{1}{b}}{\left( \min \left\{ 1,\frac{1-\theta }{2\theta }\right\} -1\right) \frac{1}{b^{2}}+\frac{1}{b}+\left( 1-\frac{1}{b}\right) ^{2}}\nonumber \\&\le l_{0}\left( \frac{1-\theta }{\theta }\right) \frac{\frac{1}{b}}{1-\frac{1}{b}}\nonumber \\&= \left( \frac{1-\theta }{\theta }\right) ^{2}\frac{b}{(b-1)^{2}}=:l_{1} \end{aligned}$$

and

$$\begin{aligned}&\lim _{k\rightarrow \infty } c_{3k}c_{3k+1}c_{3k+2}\nonumber \\&\quad = c_{1,\infty }\left( \frac{1-\theta }{\theta }\right) \frac{\left( \min \left\{ 1,\frac{2\theta }{1-\theta }\right\} -1\right) \frac{1}{b^{2}}+\frac{1}{b}}{\left( \min \left\{ 1,\frac{1-\theta }{2\theta }\right\} -1\right) \frac{1}{b^{2}}+\frac{1}{b}}\nonumber \\&\quad \le l_{1}\left( \frac{1-\theta }{\theta }\right) \frac{\frac{1}{b}}{\frac{1}{b}\left( 1-\frac{1}{b}\right) }\nonumber \\&\quad = \left( \frac{1-\theta }{\theta }\right) ^{3}\frac{b^{2}}{\left( b-1\right) ^{3}}=:l_{2}. \end{aligned}$$

Therefore, for any $\delta >0$ there exists $k_{0}\in {\mathbb {N}}^{+}$, such that whenever $k\ge k_{0}+1$

$$\begin{aligned} K&:= \prod _{j=k_{0}}^{k-1}c_{3j}c_{3j+1}c_{3j+2}\nonumber \\&< (l_{2}+\delta )^{k-k_{0}}, \end{aligned}$$

implying

$$\begin{aligned}&Kc_{3k}<(l_{2}+\delta )^{k-k_{0}}(l_{0}+\delta ),\nonumber \\&\quad Kc_{3k}c_{3k+1}<(l_{2}+\delta )^{k-k_{0}}(l_{1}+\delta )\quad \text {and}\nonumber \\&\quad Kc_{3k}c_{3k+1}c_{3k+2}<(l_{2}+\delta )^{k-k_{0}}(l_{2}+\delta ). \end{aligned}$$

Hence, for $i\in \lbrace 0,1,2\rbrace$ and some $C>0$

$$\begin{aligned} \prod _{j=2}^{3k+i}c_{j}&< C(l_{2}+\delta )^{k}. \end{aligned}$$

Let $a_{m}:=\prod _{j=2}^{m}c_{j}$, then a sufficient condition for the series $\sum _{m=2}^{\infty }a_{m}$ to converge, implying a transient chain according to Proposition 5.1, is $l_{2}<1$. This is the case for $b\ge 3+\left( \frac{1-\theta }{\theta }\right) ^{3}$, since

$$\begin{aligned} 1-l_{2}&= 1-\left( \frac{1-\theta }{\theta }\right) ^{3}\frac{b^{2}}{\left( b-1\right) ^{3}}\nonumber \\&= \frac{b^{2}}{\left( b-1\right) ^{3}}\left( \frac{\left( b-1\right) ^{3}}{b^{2}}-\left( \frac{1-\theta }{\theta }\right) ^{3}\right) \nonumber \\&= \frac{b^{2}}{\left( b-1\right) ^{3}}\left( b-3+\frac{3}{b}-\frac{1}{b^{2}}-\left( \frac{1-\theta }{\theta }\right) ^{3}\right) \nonumber \\&\quad > \frac{b^{2}}{\left( b-1\right) ^{3}}\left( b-3-\left( \frac{1-\theta }{\theta }\right) ^{3}\right) \nonumber \\&\ge 0. \end{aligned}$$

Hence, the resulting noisy chain is transient if $b\ge 3+\left( \frac{1-\theta }{\theta }\right) ^{3}$, for any $\theta \in (0,1)$. $\square$

### Section 3

#### *Proof of Lemma 3.1*

For any $$\delta >0$$

$$\begin{aligned}&{\mathbb {P}}\left[ \frac{W_{z,N}}{W_{x,N}}\le 1-\delta \right] \nonumber \\&\quad \le {\mathbb {P}}\left[ W_{x,N}\ge 1+\frac{\delta }{2}\right] + {\mathbb {P}}\left[ W_{z,N}\le 1-\frac{\delta }{2}\right] \nonumber \\&\quad \le {\mathbb {P}}\left[ \Big \vert W_{x,N}-1 \Big \vert \ge \frac{\delta }{2}\right] + {\mathbb {P}}\left[ \Big \vert W_{z,N}-1 \Big \vert \ge \frac{\delta }{2}\right] \nonumber \\&\quad \le 2\sup _{x\in \mathcal {X}}{\mathbb {P}}_{Q_{x,N}}\left[ \Big \vert W_{x,N}-1\Big \vert \ge \frac{\delta }{2}\right] . \end{aligned}$$

$$\square $$

#### *Proof of Lemma 3.2*

Using the inequality

$$\begin{aligned} \min \left\{ 1,ab\right\} \ge \min \left\{ 1,a\right\} \min \left\{ 1,b\right\} , \quad \text {for}\; a,b\ge 0, \end{aligned}$$

and applying Markov’s inequality with $\delta >0$,

$$\begin{aligned} \tilde{\rho }_{N}(x)&= 1- \int _{\mathcal {X}} q(x,dz) \tilde{\alpha }_N(x,z) \\&\le 1-\int _{\mathcal {X}}q(x,dz)\alpha (x,z){\mathbb {E}}\left[ \min \left\{ 1,\frac{W_{z,N}}{W_{x,N}}\right\} \right] \\&\le 1-\left( 1-\delta \right) \\&\quad \times \int _{\mathcal {X}}q(x,dz)\alpha (x,z){\mathbb {P}}\,\left[ \min \left\{ 1,\frac{W_{z,N}}{W_{x,N}}\right\} >1-\delta \right] \\&= 1-\left( 1-\delta \right) \int _{\mathcal {X}}q(x,dz)\alpha (x,z)+\left( 1-\delta \right) \\&\quad \times \int _{\mathcal {X}}q(x,dz)\alpha (x,z){\mathbb {P}}\left[ \frac{W_{z,N}}{W_{x,N}}\le 1-\delta \right] \\&\le 1-\left( 1-\delta \right) \left( 1-\rho (x)\right) \\&\quad +\int _{\mathcal {X}}q(x,dz)\alpha (x,z){\mathbb {P}}\left[ \frac{W_{z,N}}{W_{x,N}}\le 1-\delta \right] . \end{aligned}$$

Finally, using Lemma 3.1

$$\begin{aligned} \tilde{\rho }_{N}(x)&\le \rho (x)+\delta \left( 1-\rho (x)\right) \nonumber \\&\quad +2\sup _{x\in \mathcal {X}}{\mathbb {P}}\left[ \big \vert W_{x,N}-1\big \vert \ge \frac{\delta }{2}\right] \left( 1-\rho (x)\right) \nonumber \\&\le \rho (x)+\delta +2\sup _{x\in \mathcal {X}}{\mathbb {P}}\left[ \big \vert W_{x,N}-1\big \vert \ge \frac{\delta }{2}\right] . \end{aligned}$$

$$\square $$

#### *Proof of Lemma 3.3*

For the first claim apply Jensen’s inequality and the fact that

$$\begin{aligned} \min \left\{ 1,ab\right\} \le \min \left\{ 1,a\right\} b, \quad \text {for }\; a\ge 0 \;\text {and}\; b\ge 1, \end{aligned}$$

hence

$$\begin{aligned} \tilde{\alpha }_{N}(x,z)&\le \min \left\{ 1,\frac{\pi (z)q(z,x)}{\pi (x)q(x,z)}{\mathbb {E}}\left[ \frac{W_{z,N}}{W_{x,N}}\right] \right\} \nonumber \\&\le \alpha (x,z){\mathbb {E}}\left[ W_{x,N}^{-1}\right] {\mathbb {E}}\left[ W_{z,N}\right] . \end{aligned}$$

$$\square $$

#### *Proof of Lemma 3.4*

Using the inequality

$$\begin{aligned}&\min \left\{ 1,ab\right\} \le \min \left\{ 1,a\right\} b, \quad \text {for}\;a\ge 0\; \text {and}\; b\ge 1,\\&\tilde{\alpha }_{N} (x,z)\nonumber \\&\quad = {\mathbb {E}}\left[ {\bar{\alpha }}_{N}(x,W_{x,N};z,W_{z,N})\mathbbm {1}_{\left\{ \frac{W_{z,N}}{W_{x,N}}<1+\eta \right\} }\right] \nonumber \\&\qquad +{\mathbb {E}}\left[ {\bar{\alpha }}_{N}(x,W_{x,N};z,W_{z,N})\mathbbm {1}_{\left\{ \frac{W_{z,N}}{W_{x,N}}\ge 1+\eta \right\} }\right] \nonumber \\&\quad \le \alpha (x,z)\left( 1+\eta \right) {\mathbb {P}}\left[ \frac{W_{z,N}}{W_{x,N}}<1+\eta \right] \nonumber \\&\qquad +{\mathbb {P}}\left[ \frac{W_{z,N}}{W_{x,N}}\ge 1+\eta \right] \nonumber \\&\quad \le \alpha (x,z)+\eta +{\mathbb {P}}\left[ \frac{W_{z,N}}{W_{x,N}}\ge 1+\eta \right] , \end{aligned}$$

Notice that

$$\begin{aligned} {\mathbb {P}}\left[ \frac{W_{z,N}}{W_{x,N}}\ge 1+\eta \right] ={\mathbb {P}}\left[ \frac{W_{x,N}}{W_{z,N}}\le \frac{1}{1+\eta } \right] , \end{aligned}$$

then applying Lemma 3.1 taking $\delta =\frac{\eta }{1+\eta }$.

$$\begin{aligned} \tilde{\alpha }_{N} (x,z)&\le \alpha (x,z)+\eta \nonumber \\&\quad +2\sup _{x\in \mathcal {X}}{\mathbb {P}}\left[ \Big \vert W_{x,N}-1\Big \vert \ge \frac{\eta }{2\left( 1+\eta \right) }\right] . \end{aligned}$$

$$\square $$

#### *Proof of Proposition 3.1*

Taking $$V=\pi ^{-s}$$, where $$0<s<\min \left\{ 1,\frac{a}{L}\right\} $$,

$$\begin{aligned} \frac{qV(x)}{V(x)}&= \int _{\mathcal {X}}\frac{V(z)}{V(x)}q(x,dz)\nonumber \\&= \int _{\mathcal {X}}\left( \frac{\pi (x)}{\pi (z)}\right) ^{s}q(x,dz)\nonumber \\&\le \int _{{\mathbb {R}}^{d}}\exp \left\{ a\Vert z-x\Vert \right\} q(\Vert z-x\Vert )dz. \end{aligned}$$

Finally, using the transformation $u=z-x$,

$$\begin{aligned} \frac{qV(x)}{V(x)} \le \int _{{\mathbb {R}}^{d}}\exp \left\{ a\Vert u\Vert \right\} q(\Vert u\Vert )du, \end{aligned}$$

which implies (P1*). $$\square $$

#### *Proof of Proposition 3.2*

By properties of the arithmetic and harmonic means

$$\begin{aligned} \frac{1}{N}\sum _{k=1}^{N}\frac{1}{W_{x}^{(k)}}-\frac{N}{\sum _{k=1}^{N}W_{x}^{(k)}} \ge 0, \end{aligned}$$

which implies, by Jensen’s inequality,

$$\begin{aligned} {\mathbb {E}}\left[ \frac{1}{N}\sum _{k=1}^{N}\frac{1}{W_{x}^{(k)}}-\frac{N}{\sum _{k=1}^{N}W_{x}^{(k)}}\right]&\le {\mathbb {E}}\left[ W_{x}^{-1}\right] -1. \end{aligned}$$

Then, using Fatou’s lemma and the law of large numbers

$$\begin{aligned} {\mathbb {E}}\left[ W_{x}^{-1}\right] -1&\ge \limsup _{N\rightarrow \infty }{\mathbb {E}}\left[ \frac{1}{N}\sum _{k=1}^{N}\frac{1}{W_{x}^{(k)}}-W_{x,N}^{-1}\right] \nonumber \\&\ge \liminf _{N\rightarrow \infty }{\mathbb {E}}\left[ \frac{1}{N}\sum _{k=1}^{N}\frac{1}{W_{x}^{(k)}}-W_{x,N}^{-1}\right] \nonumber \\&\ge {\mathbb {E}}\left[ \liminf _{N\rightarrow \infty } \left( \frac{1}{N}\sum _{k=1}^{N}\frac{1}{W_{x}^{(k)}}\right) \right. \nonumber \\&\quad \left. -\limsup _{N\rightarrow \infty }W_{x,N}^{-1}\right] \ge {\mathbb {E}}\left[ W_{x}^{-1}\right] -1, \end{aligned}$$

hence

34 $$\begin{aligned} \lim _{N\rightarrow \infty } {\mathbb {E}}\left[ \frac{1}{N}\sum _{k=1}^{N}\frac{1}{W_{x}^{(k)}}-W_{x,N}^{-1}\right] =E\left[ W_{x}^{-1}\right] -1. \end{aligned}$$

Finally, since

$$\begin{aligned} {\mathbb {E}}\left[ \frac{1}{N}\sum _{k=1}^{N}\frac{1}{W_{x}^{(k)}}-W_{x,N}^{-1}\right]&= E\left[ W_{x}^{-1}\right] -E\left[ W_{x,N}^{-1}\right] , \end{aligned}$$

the expression in (34) becomes

$$\begin{aligned} \lim _{N\rightarrow \infty } {\mathbb {E}}\left[ W_{x,N}^{-1}\right] =1. \end{aligned}$$

$$\square $$

#### *Proof of Lemma 3.5*

The proof of (*i*) is motivated by Piegorsch and Casella (1985, Theorem 2.1) and Khuri and Casella (2002, Theorem 3), however the existence of a density function is not assumed here. Since $Z^{-p}$ is positive,

$$\begin{aligned} {\mathbb {E}}\left[ Z^{-p} \right]&= \int _{{\mathbb {R}}^+}{\mathbb {P}}\left[ Z^{-p} \ge z \right] dz\nonumber \\&\le \frac{1}{\gamma ^p} + \int _{\left( \gamma ^{-p}, \infty \right) }{\mathbb {P}}\left[ Z^{-p} \ge z \right] dz\nonumber \\&= \frac{1}{\gamma ^p} + \int _{\left( 0, \gamma \right) } pu^{-p-1} {\mathbb {P}}\left[ Z \le u \right] du\nonumber \\&\le \frac{1}{\gamma ^p} + pM\frac{\gamma ^{\alpha -p}}{\alpha -p}. \end{aligned}$$

For part (ii), since the random variables $\left\{ Z_{i}\right\}$ are positive, then for any $z>0$

$$\begin{aligned} {\mathbb {P}}\left[ \sum _{i=1}^{N}Z_{i}\le z\right] ={\mathbb {P}}\left[ \sum _{i=1}^{N}Z_{i}\le z,\max _{i\in \{1,\ldots ,N\}}\left\{ Z_{i}\right\} \le z\right] . \end{aligned}$$

Therefore, for $$z\in (0,\gamma )$$

$$\begin{aligned} {\mathbb {P}}\left[ \sum _{i=1}^{N}Z_{i}\le z\right]&\le {\mathbb {P}}\left[ \max _{i\in \{1,\ldots ,N\}}\left\{ Z_{i}\right\} \le z\right] \nonumber \\&=\prod _{i=1}^{N}{\mathbb {P}}\left[ Z_{i}\le z\right] \nonumber \\&\le \prod _{i=1}^{N}M_{i}z^{\sum _{i=1}^{N}\alpha _{i}}. \end{aligned}$$

Part (iii) can be seen as a consequence of $W_{x,N}$ and $W_{x,N+1}$ being convex ordered and $g(x)=x^{-p}$ being a convex function for $x>0$ and $p\ge 0$, (see, e.g., Andrieu and Vihola 2014). We provide a self-contained proof by defining for $j\in \{1,\ldots ,N+1\}$

$$\begin{aligned} S_{x,N}^{(j)}:=\frac{1}{N} \sum _{k=1,k\ne j}^{N+1} W_{x}^{(k)}, \end{aligned}$$

and we have

$$\begin{aligned} W_{x,N+1}=\frac{1}{N+1} \sum _{j=1}^{N+1} S_{x,N}^{(j)} \end{aligned}$$

and since the arithmetic mean is greater than or equal to the geometric mean

$$\begin{aligned} W_{x,N+1}\ge \left( \prod _{j=1}^{N+1} S_{x,N}^{(j)} \right) ^{\frac{1}{N+1}}. \end{aligned}$$

This implies for $p>0$

$$\begin{aligned} {\mathbb {E}}\left[ W_{x,N+1}^{-p} \right]&\le {\mathbb {E}}\left[ \left( \prod _{j=1}^{N+1} S_{x,N}^{(j)} \right) ^{-\frac{p}{N+1}} \right] \nonumber \\&\le \prod _{j=1}^{N+1} \left( {\mathbb {E}}\left[ \left( S_{x,N}^{(j)} \right) ^{-p} \right] \right) ^{\frac{1}{N+1}}\nonumber \\&= {\mathbb {E}}\left[ \left( S_{x,N}^{(1)} \right) ^{-p} \right] \nonumber \\&= {\mathbb {E}}\left[ W_{x,N}^{-p} \right] , \end{aligned}$$

where Hölder’s inequality has been used and the fact that the random variables $\left\{ S_{x,N}^{(j)} : j \in {1,\ldots ,N+1} \right\}$ are identically distributed according to $Q_{x,N}$.

For part (iv), let $M_{\gamma }=\sup _{y\in [\gamma ,\infty )}|g(y)|$ and due to continuity at $y=1$, for any $\varepsilon >0$ there exists a $\delta >0$ such that

$$\begin{aligned}&{\mathbb {E}}\left[ \big \vert g{\left( W_{x,N}\right) -g(1)\big \vert \mathbbm {1}_{W_{x,N}\in [\gamma ,\infty )}}\right] \nonumber \\&\quad \le 2M_{\gamma }{\mathbb {P}}\left[ \gamma \le W_{x,N}\le 1-\delta \right] +2M_{\gamma }{\mathbb {P}}\left[ 1+\delta \le W_{x,N}\right] \nonumber \\&\qquad +{\mathbb {E}}\left[ \big \vert g\left( W_{x,N}\right) -g(1)\big \vert \mathbbm {1}_{W_{x,N}\in \left( 1-\delta ,1+\delta \right) }\right] \nonumber \\&\quad \le 2M_{\gamma }{\mathbb {P}}\left[ \big \vert W_{x,N}-1\big \vert \ge \delta \right] +\varepsilon {\mathbb {P}}\left[ \big \vert W_{x,N}-1\big \vert < \delta \right] . \end{aligned}$$

Therefore, for fixed $\varepsilon$ and by (W1)

$$\begin{aligned}&\lim _{N\rightarrow \infty }\sup _{x\in \mathcal {X}} {\mathbb {E}} \left[ \big \vert g\left( W_{x,N}\right) -g(1)\big \vert \mathbbm {1}_{W_{x,N}\in [\gamma ,\infty )}\right] \nonumber \\&\quad \le 2M_{\gamma }\lim _{N\rightarrow \infty }\sup _{x\in \mathcal {X}}{\mathbb {P}}\left[ \big \vert W_{x,N}-1\big \vert \ge \delta \right] +\varepsilon \nonumber \\&\quad \le \varepsilon , \end{aligned}$$

obtaining the result since $\varepsilon$ can be picked arbitrarily small.$\square$

#### *Proof of Proposition 3.4*

First notice that if $l<\infty$ then $l\ge 1$. To see this, define

$$\begin{aligned} a_{m} := \frac{\varepsilon _{m-1}}{\varepsilon _{m}}, \end{aligned}$$

then for fixed $\delta >0$, there exists $M\in {\mathbb {N}}$ such that for $m\ge M$

$$\begin{aligned} a_{m} < l+\delta . \end{aligned}$$

Then, for $$m\ge M$$

$$\begin{aligned} \frac{\varepsilon _{1}}{\varepsilon _{m}}&= \prod _{j=2}^{m}a_{j}\nonumber \\&< \left( l+\delta \right) ^{m-M}\frac{\varepsilon _{1}}{\varepsilon _{M}}, \end{aligned}$$

and because $\varepsilon _{m}\rightarrow 0$, it is clear that $\left( l+\delta \right) ^{m}\rightarrow \infty$ as $m\rightarrow \infty$. Therefore, $l+\delta >1$ and since $\delta$ can be taken arbitrarily small, it is true that $l\ge 1$.

Now, for weights as in (21) and using a simple random walk proposal, the noisy acceptance probability can be expressed as

35 $$\begin{aligned}&\tilde{\alpha }_{N} (m,m-1)\nonumber \\&\quad = \sum _{j=0}^{N}\sum _{k=0}^{N}\min \left\{ 1,\frac{2\theta }{1-\theta }\frac{b_{m-1}j+\left( N-j\right) \varepsilon _{m-1}}{b_{m}k+\left( N-k\right) \varepsilon _{m}}\right\} \left( {\begin{array}{c}N\\ j\end{array}}\right) \nonumber \\&\qquad \times \left( {\begin{array}{c}N\\ k\end{array}}\right) \left( s_{m-1}\right) ^{j}\left( s_{m}\right) ^{k}\left( 1-s_{m-1}\right) ^{N-j}\left( 1-s_{m}\right) ^{N-k} \end{aligned}$$

and

36 $$\begin{aligned}&\tilde{\alpha }_{N} (m,m+1)\nonumber \\&\quad = \sum _{j=0}^{N}\sum _{k=0}^{N}\min \left\{ 1,\frac{1-\theta }{2\theta }\frac{b_{m+1}j+\left( N-j\right) \varepsilon _{m+1}}{b_{m}k+\left( N-k\right) \varepsilon _{m}}\right\} \left( {\begin{array}{c}N\\ j\end{array}}\right) \nonumber \\&\qquad \times \left( {\begin{array}{c}N\\ k\end{array}}\right) \left( s_{m+1}\right) ^{j}\left( s_{m}\right) ^{k}\left( 1-s_{m+1}\right) ^{N-j}\left( 1-s_{m}\right) ^{N-k}. \end{aligned}$$

Since $b_{m}\rightarrow \infty$, then $s_{m}\rightarrow 0$ as $m\rightarrow \infty$; therefore, any term in (35) and (36), for which $j+k\ne 0$, tends to zero as $m\rightarrow \infty$. Hence,

$$\begin{aligned}&\tilde{\alpha }_{N} (m,m-1)\nonumber \\&\quad = \min \left\{ 1,\frac{2\theta }{1-\theta }\times \frac{\varepsilon _{m-1}}{\varepsilon _{m}}\right\} \nonumber \\&\qquad \times \left( 1-s_{m-1}\right) ^{N}\left( 1-s_{m}\right) ^{N}+o(1) \end{aligned}$$

and

$$\begin{aligned}&\tilde{\alpha }_{N} (m,m+1)\nonumber \\&\quad = \min \left\{ 1,\frac{1-\theta }{2\theta }\times \frac{\varepsilon _{m+1}}{\varepsilon _{m}}\right\} \nonumber \\&\qquad \times \left( 1-s_{m+1}\right) ^{N}\left( 1-s_{m}\right) ^{N}+o(1), \end{aligned}$$

implying,

37 $$\begin{aligned}&\lim _{m\rightarrow \infty } \frac{{\tilde{P}}_{N}\left( m,\left\{ m-1\right\} \right) }{{\tilde{P}}_{N}\left( m,\left\{ m+1\right\} \right) }\nonumber \\&\quad = \frac{\left( 1-\theta \right) \lim _{m\rightarrow \infty }\min \left\{ 1,\frac{2\theta }{1-\theta }\times \frac{\varepsilon _{m-1}}{\varepsilon _{m}}\right\} }{\theta \lim _{m\rightarrow \infty }\min \left\{ 1,\frac{1-\theta }{2\theta }\times \frac{\varepsilon _{m+1}}{\varepsilon _{m}}\right\} }. \end{aligned}$$

If $l=+\infty$, (37) tends to $+\infty$, whereas if $l<\infty$

$$\begin{aligned} \lim _{m\rightarrow \infty }\frac{{\tilde{P}}_{N} \left( m,\left\{ m-1\right\} \right) }{{\tilde{P}}_{N} \left( m,\left\{ m+1\right\} \right) }&= 2l\frac{\min \left\{ 1-\theta ,2\theta l\right\} }{\min \left\{ 2\theta l,1-\theta \right\} }\nonumber \\&\ge 2. \end{aligned}$$

In any case, this implies

$$\begin{aligned} \lim _{m\rightarrow \infty }{\tilde{P}}_{N}\left( m,\left\{ m-1\right\} \right)&\ge 2\lim _{m\rightarrow \infty }{\tilde{P}}_{N}\left( m,\left\{ m+1\right\} \right) , \end{aligned}$$

and since

$$\begin{aligned} \lim _{m\rightarrow \infty }{\tilde{P}}_{N}\left( m,\left\{ m-1\right\} \right)&= \min \left\{ 1-\theta ,2\theta l\right\} \nonumber \\&> 0, \end{aligned}$$

the noisy chain is geometrically ergodic according to Proposition Proposition 5.1. $\square$

#### *Proof of Proposition 3.5*

Noting that

$$\begin{aligned} \frac{\varepsilon _{m-1}}{\varepsilon _{m}}&\in {\left\{ \begin{array}{ll} \begin{array}{l} O\left( m^{2}\right) \\ O\left( m^{-1}\right) \end{array}\quad \text {if} &{} \begin{array}{l} m\ (\text {mod}\ 3)=0,\\ m\ (\text {mod}\ 3)\in \{1,2\}, \end{array}\end{array}\right. }\\ \text {and}\quad \frac{\varepsilon _{m+1}}{\varepsilon _{m}}&\in {\left\{ \begin{array}{ll} \begin{array}{l} O\left( m^{-2}\right) \\ O\left( m\right) \end{array}\quad \text {if} &{} \begin{array}{l} m\ (\text {mod}\ 3)=2,\\ m\ (\text {mod}\ 3)\in \{0,1\}, \end{array}\end{array}\right. } \end{aligned}$$

expressions in (35) and (36) become

$$\begin{aligned} \tilde{\alpha }_{N}(m,m-1)&= \left( 1-s_{m-1}\right) ^{N}\left( 1-s_{m}\right) ^{N}\mathbbm {1}_{\left\{ m\ (\text {mod}\ 3)=0\right\} }\nonumber \\&\quad +O(m^{-1}) \end{aligned}$$

and

$$\begin{aligned} \tilde{\alpha }_{N}(m,m+1)&= \left( 1-s_{m+1}\right) ^{N}\left( 1-s_{m}\right) ^{N}\mathbbm {1}_{\left\{ m\ (\text {mod}\ 3)=0,1\right\} }\nonumber \\&\quad +O\left( m^{-1}\right) . \end{aligned}$$

Therefore,

$$\begin{aligned}&{\tilde{P}}_{N}\left( m,\left\{ m-1\right\} \right) / {\tilde{P}}_{N}\left( m,\left\{ m+1\right\} \right) \nonumber \\&\quad = \left( \frac{1-\theta }{\theta }\right) \frac{\left( 1-s_{m-1}\right) ^{N}+O(m^{-1})}{\left( 1-s_{m+1}\right) ^{N}+O(m^{-1})}\mathbbm {1}_{\left\{ m\ (\text {mod}\ 3)=0\right\} }\nonumber \\&\qquad +O\left( m^{-1}\right) \mathbbm {1}_{\left\{ m\ (\text {mod}\ 3)=1\right\} }\nonumber \\&\qquad +O(1)\mathbbm {1}_{\left\{ m\ (\text {mod}\ 3)=2\right\} }, \end{aligned}$$

implying there exists $C\in {\mathbb {R}}^{+}$ such that for $j=0,2$

$$\begin{aligned} \lim _{k\rightarrow \infty }\frac{{\tilde{P}}_{N}\left( 3k+j,\left\{ 3k+j-1\right\} \right) }{{\tilde{P}}_{N}\left( 3k+j,\left\{ 3k+j+1\right\} \right) } \le C, \end{aligned}$$

and

$$\begin{aligned} \lim _{k\rightarrow \infty }\frac{{\tilde{P}}_{N}\left( 3k+1,\left\{ 3k\right\} \right) }{{\tilde{P}}_{N}\left( 3k+1,\left\{ 3k+2\right\} \right) } = 0. \end{aligned}$$

Then, for fixed $\delta >0$ there exists $k_{0}\in {\mathbb {N}}^{+}$ such that whenever $k\ge k_{0}$

$$\begin{aligned} \frac{{\tilde{P}}_{N}\left( 3k+j,\left\{ 3k+j-1\right\} \right) }{{\tilde{P}}_{N}\left( 3k+j,\left\{ 3k+j+1\right\} \right) } < C+\delta ,\quad \text {for}\;j=0,2 \end{aligned}$$

and

$$\begin{aligned} \frac{{\tilde{P}}_{N}\left( 3k+1,\left\{ 3k\right\} \right) }{{\tilde{P}}_{N}\left( 3k+1,\left\{ 3k+2\right\} \right) } < \delta . \end{aligned}$$

Let

$$\begin{aligned} c_{m} := \frac{{\tilde{P}}_{N}(m,\left\{ m-1\right\} )}{{\tilde{P}}_{N}(m,\left\{ m+1\right\} )}, \end{aligned}$$

then for $$k\ge k_{0}+1$$

$$\begin{aligned} \prod _{j=2}^{3k+1}c_{j}&= \prod _{j=1}^{k}c_{3j-1}c_{3j}c_{3j+1}\nonumber \\&\le \left( \left( C+\delta \right) ^{2}\delta \right) ^{k-k_{0}}\prod _{j=1}^{k_{0}}c_{3j-1}c_{3j}c_{3j+1}. \end{aligned}$$

Take $\delta$ small enough, such that $\left( C+\delta \right) ^{2}\delta <1$, hence

$$\begin{aligned}&\sum _{k=1}^{\infty }\prod _{j=2}^{3k+1}c_{j} = \sum _{k=1}^{k_{0}}\prod _{j=2}^{3k+1}c_{j}+\sum _{k=k_{0}}^{\infty }\prod _{j=2}^{3k+1}c_{j}\nonumber \\&\le \sum _{k=1}^{k_{0}}\prod _{j=2}^{3k+1}c_{j}+\prod _{j=1}^{k_{0}}c_{3j-1}c_{3j}c_{3j+1}\nonumber \\&\quad \times \sum _{k=k_{0}}^{\infty }\left( \left( C+\delta \right) ^{2}\delta \right) ^{k-k_{0}}\nonumber \\&= \sum _{k=1}^{k_{0}}\prod _{j=2}^{3k+1}c_{j}+\frac{\prod _{j=1}^{k_{0}}c_{3j-1}c_{3j}c_{3j+1}}{1-\left( C+\delta \right) ^{2}\delta }\nonumber \\&\quad < \infty . \end{aligned}$$

Similarly, it can be proved that

$$\begin{aligned} \sum _{k=0}^{\infty }\prod _{j=2}^{3k+2}c_{j} < \infty \quad \text {and}\quad \sum _{k=1}^{\infty }\prod _{j=2}^{3k}c_{j}<\infty , \end{aligned}$$

thus

$$\begin{aligned} \sum _{m=2}^{\infty }\prod _{j=2}^{m}c_{j} < \infty , \end{aligned}$$

implying the noisy chain is transient according to Proposition 5.1. $\square$

### Section 4

#### *Proof of Proposition 4.1*

From (23) and taking $\delta <\frac{1}{2}$, $\eta =\frac{\delta }{1-\delta }$

$$\begin{aligned}&\sup _{x\in \mathcal {X}}\Vert {\tilde{P}}_{N}(x,\cdot ) -P(x,\cdot )\Vert _{TV}\nonumber \\&\quad \le 3\delta +4\sup _{x\in \mathcal {X}}{\mathbb {P}}\left[ \Big \vert W_{x,N}-1\Big \vert \ge \frac{\delta }{2}\right] . \end{aligned}$$

Using Markov’s inequality

$$\begin{aligned}&\sup _{x\in \mathcal {X}}\Vert {\tilde{P}}_{N} (x,\cdot )-P(x,\cdot )\Vert _{TV}\nonumber \\&\quad \le 3\delta +4\sup _{x\in \mathcal {X}}{\mathbb {P}}\left[ \Big \vert W_{x,N}-1\Big \vert ^{1+k}\ge \left( \frac{\delta }{2}\right) ^{1+k}\right] \nonumber \\&\quad \le 3\delta +\frac{2^{3+k}}{\delta ^{1+k}}\sup _{x\in \mathcal {X}}{\mathbb {E}}\left[ \Big \vert W_{x,N}-1\Big \vert ^{1+k}\right] \nonumber \\&\quad \le 3\delta +\frac{2^{3+k}}{\delta ^{1+k}N^{k}}\sup _{x\in \mathcal {X}}{\mathbb {E}}\left[ \Big \vert W_{x}-1\Big \vert ^{1+k}\right] . \end{aligned}$$

Now, let

$$\begin{aligned} C_{k} = \sup _{x\in \mathcal {X}}{\mathbb {E}}\left[ \Big \vert W_{x}-1\Big \vert ^{1+k}\right] , \end{aligned}$$

then the convex function $f:{\mathbb {R}}^{+}\rightarrow {\mathbb {R}}^{+}$ where

$$\begin{aligned} f(s) = 3s+\frac{2^{3+k}C_k}{s^{1+k}N^{k}}, \end{aligned}$$

is minimised at

$$\begin{aligned} s_{*}&= \left( \frac{(1+k)2^{3+k}C_k}{3N^{k}}\right) ^{\frac{1}{2+k}}\nonumber \\&= O\left( N^{-\frac{k}{2+k}}\right) . \end{aligned}$$

Then,

$$\begin{aligned}&\sup _{x\in \mathcal {X}}\Vert {\tilde{P}}_{N}(x,\cdot ) -P(x,\cdot )\Vert _{TV}\nonumber \\&\quad \le f(s_{*})\nonumber \\&\quad = O\left( N^{-\frac{k}{2+k}}\right) +O\left( N^{-k+\frac{k(1+k)}{2+k}}\right) \nonumber \\&\quad = O\left( N^{-\frac{k}{2+k}}\right) . \end{aligned}$$

Applying Theorem 4.2 by taking

$$\begin{aligned} r(N) \propto N^{1-\frac{2}{2+k}} \end{aligned}$$

and noting $\log \left( N^{1-\frac{2}{2+k}} \right) \le \log (N)$, the result is obtained.

For the second claim, for a given $\varepsilon \in (0,1)$ take $k_{\varepsilon }\ge 2\left( \varepsilon ^{-1}-1\right)$ and apply the first part. $\square$
