# Supplementary material for: Stability of noisy Metropolis–Hastings
Source: Stat Comput. 2015 Nov 7;26(6):1187–211. doi: 10.1007/s11222-015-9604-3 (PMC6991990; doi:10.1007/s11222-015-9604-3)
Supplement: Supplementary file 1 — Supplementary material 1 (pdf 1719 KB) [file 11222_2015_9604_MOESM1_ESM.pdf]

Online Appendix 2

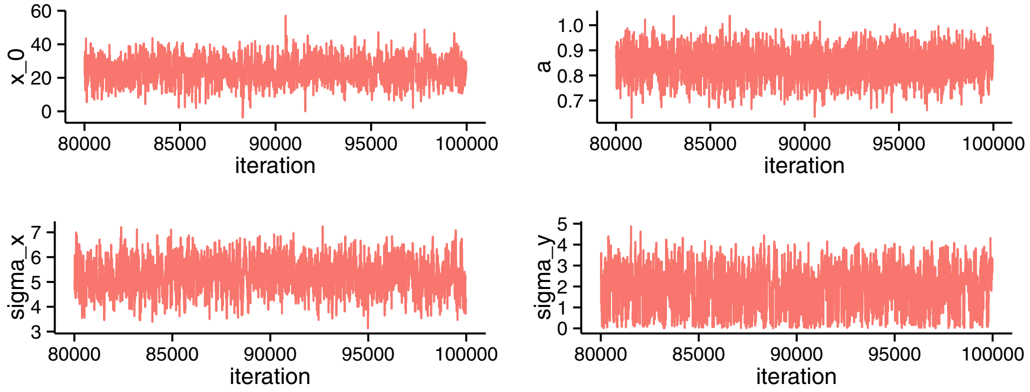

Fig. 8 Last 20,000 iterations of the marginal algorithm for the 4 parameters. The mean acceptance probability was 0.256

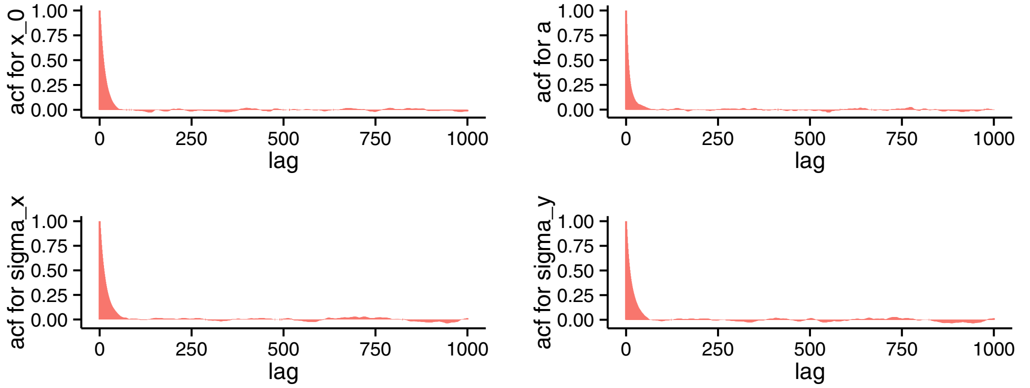

Fig. 9 Estimated autocorrelation function of the corresponding marginal chain for the 4 parameters

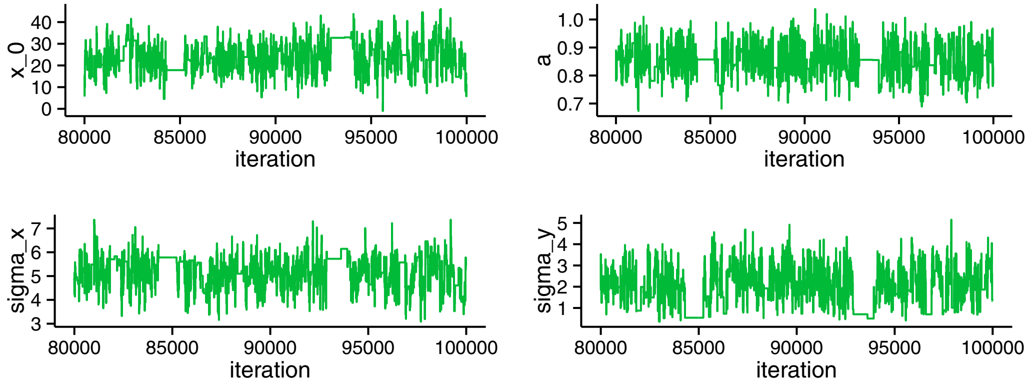

Fig. 10 Last 20,000 iterations of the pseudo-marginal algorithm for the 4 parameters when $N = 250$. The mean acceptance probability was 0.104

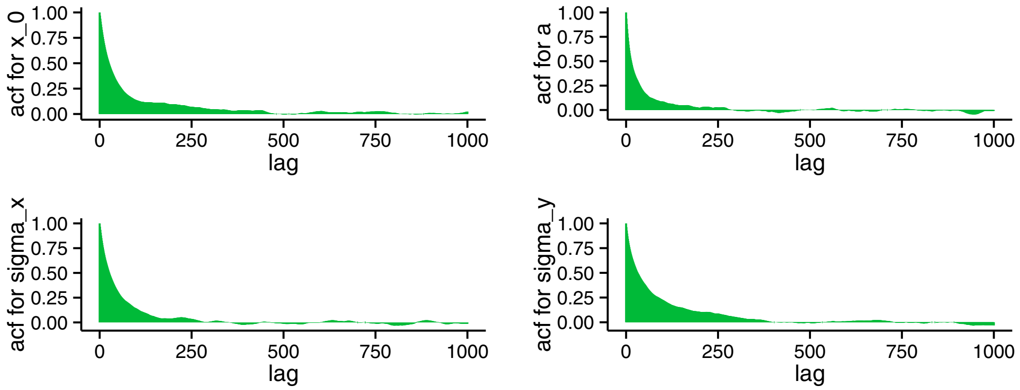

Fig. 11 Estimated autocorrelation function of the corresponding pseudo-marginal chain for the 4 parameters when $N = 250$

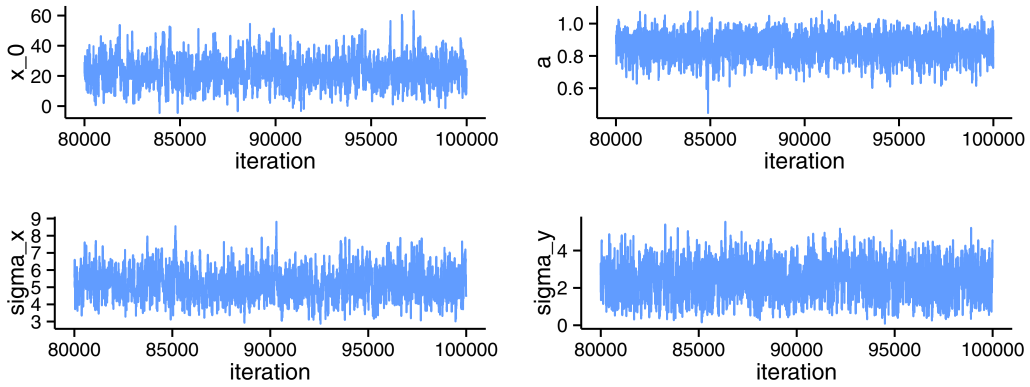

Fig. 12 Last 20,000 iterations of the pseudo-marginal algorithm for the 4 parameters when $N = 250$. The mean acceptance probability was 0.283

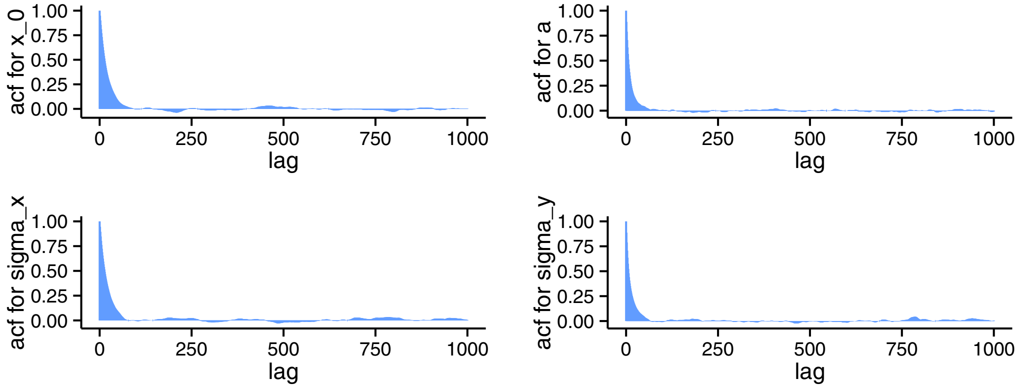

Fig. 13 Estimated autocorrelation function of the corresponding pseudo-marginal chain for the 4 parameters when $N = 250$

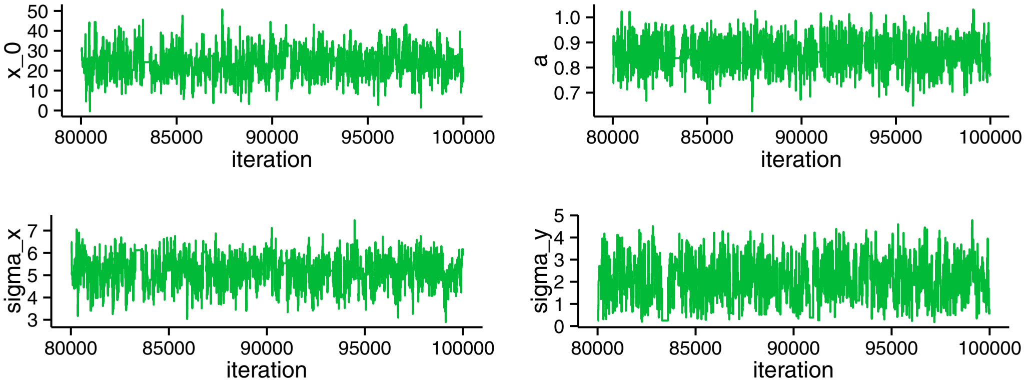

Fig. 14 Last 20,000 iterations of the pseudo-marginal algorithm for the 4 parameters when $N = 500$. The mean acceptance probability was 0.147

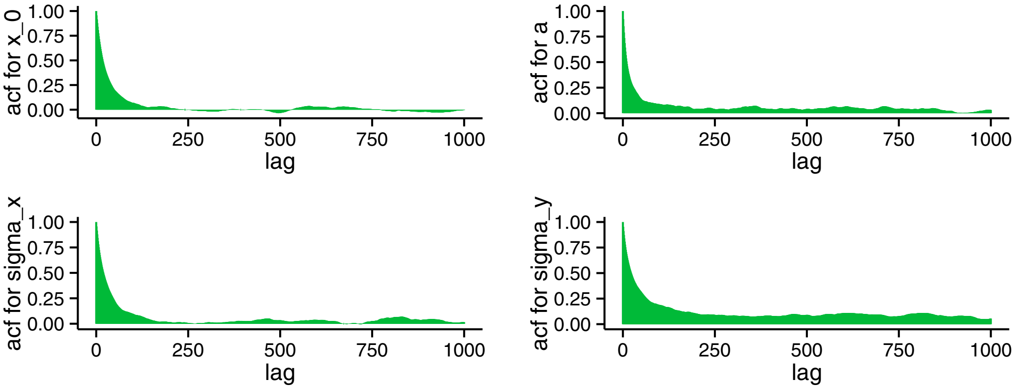

Fig. 15 Estimated autocorrelation function of the corresponding pseudo-marginal chain for the 4 parameters when $N = 500$

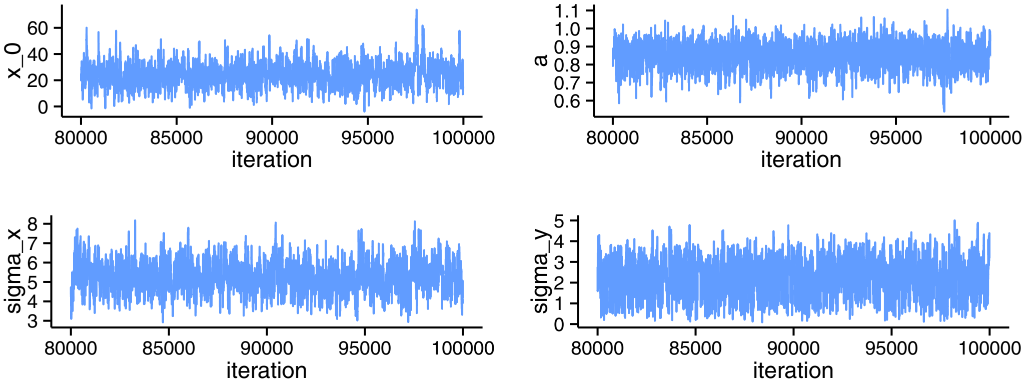

Fig. 16 Last 20,000 iterations of the pseudo-marginal algorithm for the 4 parameters when $N = 500$. The mean acceptance probability was 0.275

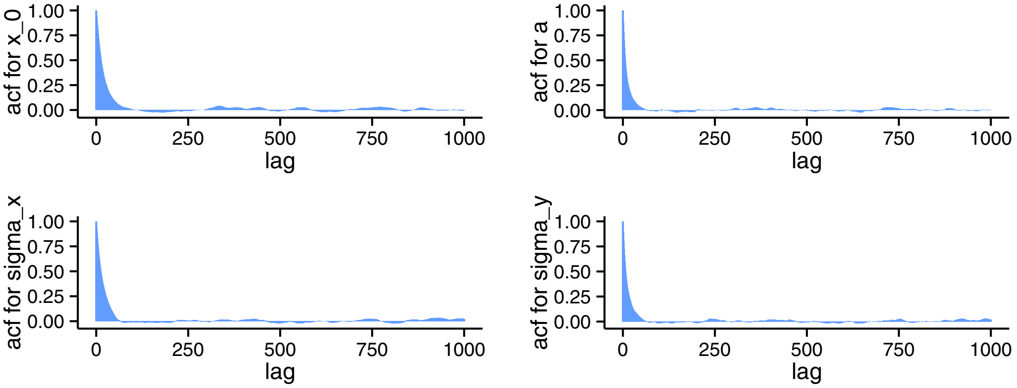

Fig. 17 Estimated autocorrelation function of the corresponding pseudo-marginal chain for the 4 parameters when $N = 500$

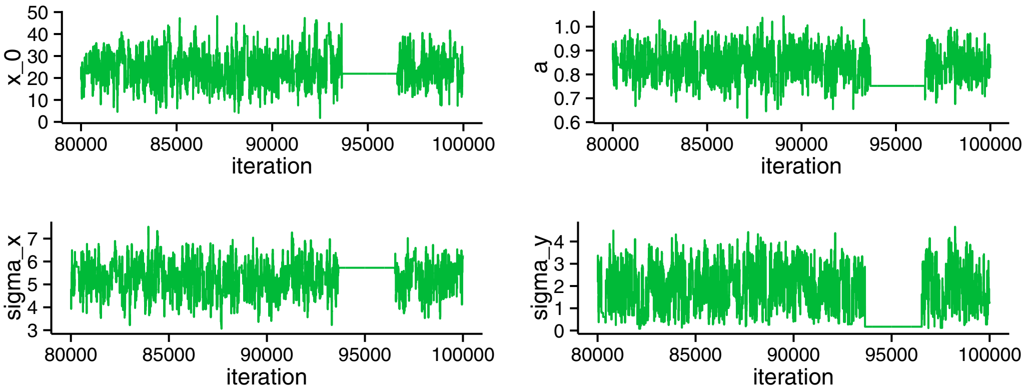

Fig. 18 Last $20,000$ iterations of the pseudo-marginal algorithm for the 4 parameters when $N = 750$. The mean acceptance probability was 0.166

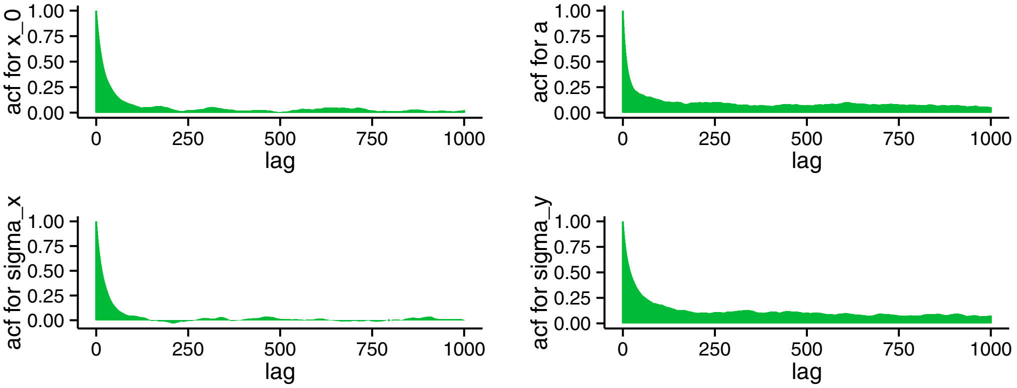

Fig. 19 Estimated autocorrelation function of the corresponding pseudo-marginal chain for the 4 parameters when $N = 750$

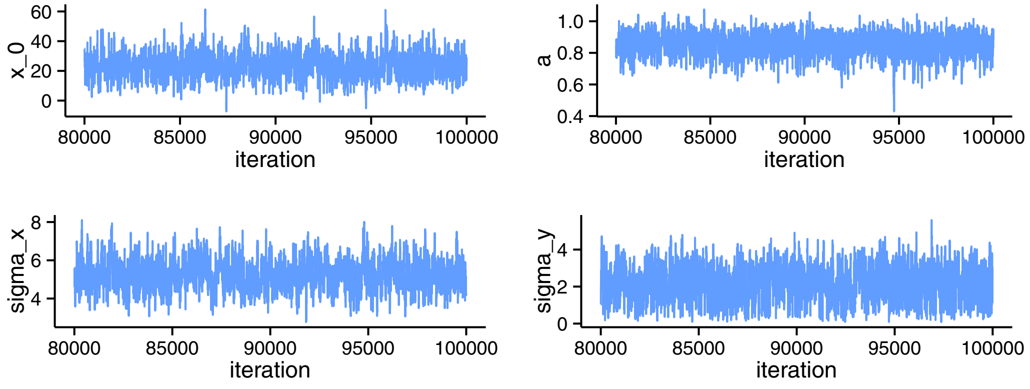

Fig. 20 Last 20,000 iterations of the pseudo-marginal algorithm for the 4 parameters when $N = 750$. The mean acceptance probability was 0.273

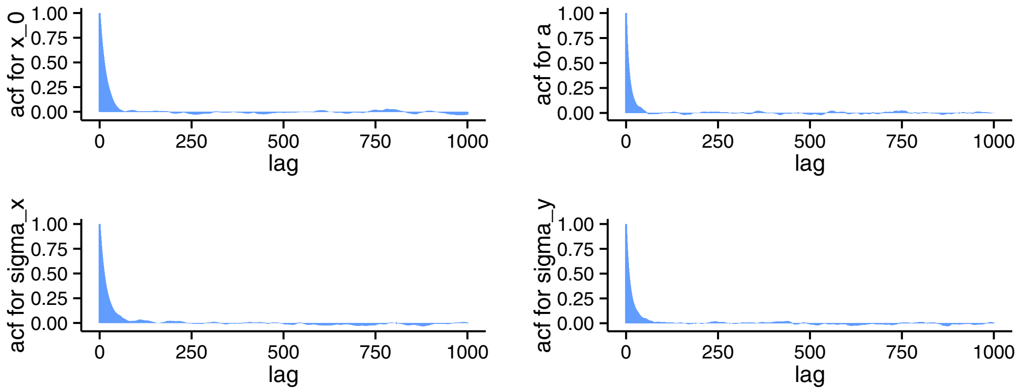

Fig. 21 Estimated autocorrelation function of the corresponding pseudo-marginal chain for the 4 parameters when $N = 750$

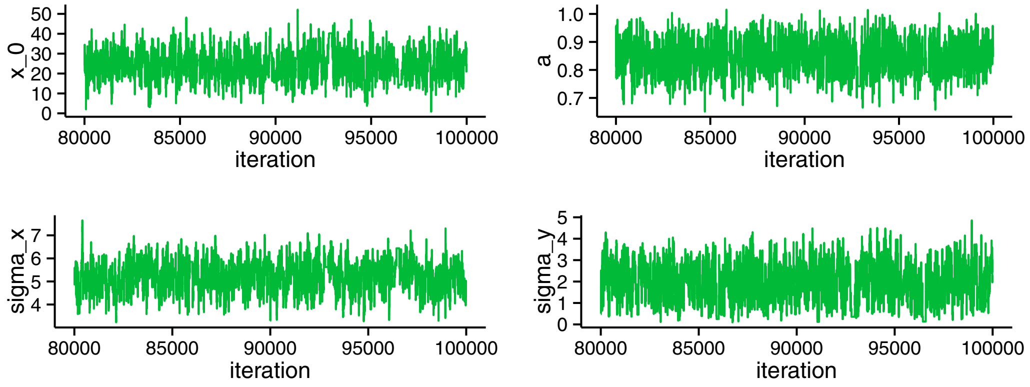

Fig. 22 Last 20,000 iterations of the pseudo-marginal algorithm for the 4 parameters when $N = 1,000$. The mean acceptance probability was 0.186

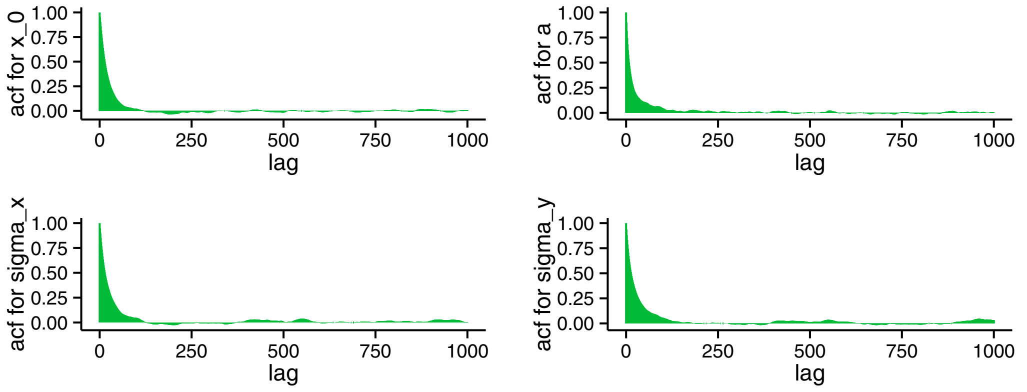

Fig. 23 Estimated autocorrelation function of the corresponding pseudo-marginal chain for the 4 parameters when $N = 1,000$

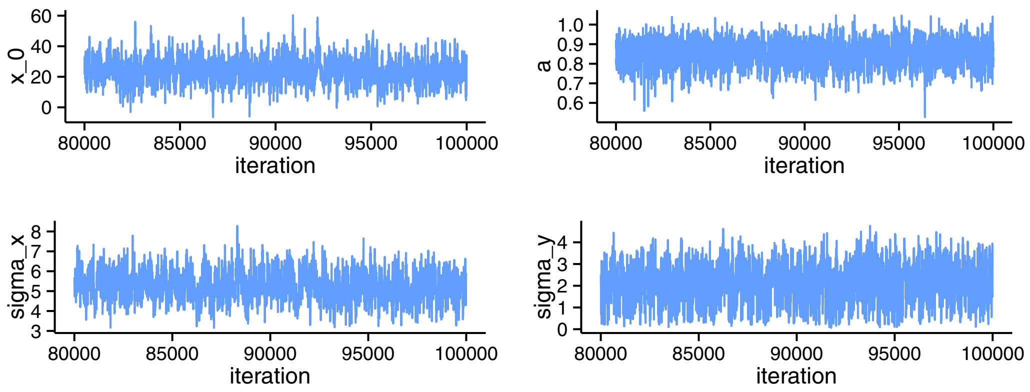

Fig. 24 Last 20,000 iterations of the pseudo-marginal algorithm for the 4 parameters when $N = 1,000$. The mean acceptance probability was 0.272

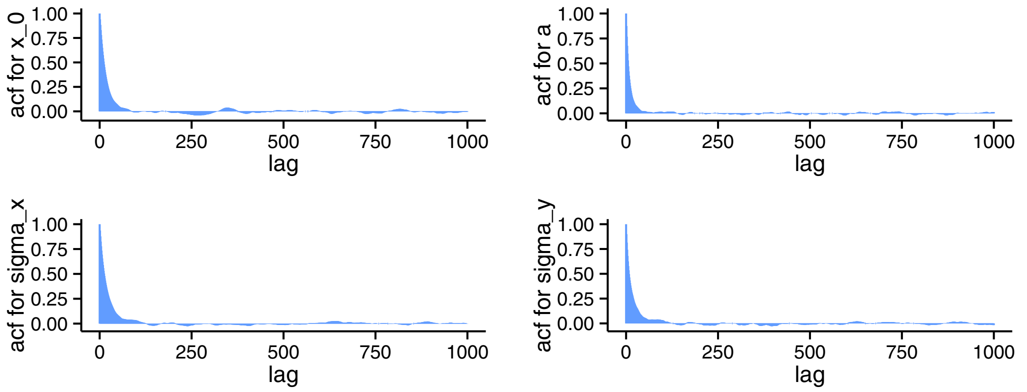

Fig. 25 Estimated autocorrelation function of the corresponding pseudo-marginal chain for the 4 parameters when $N = 1,000$

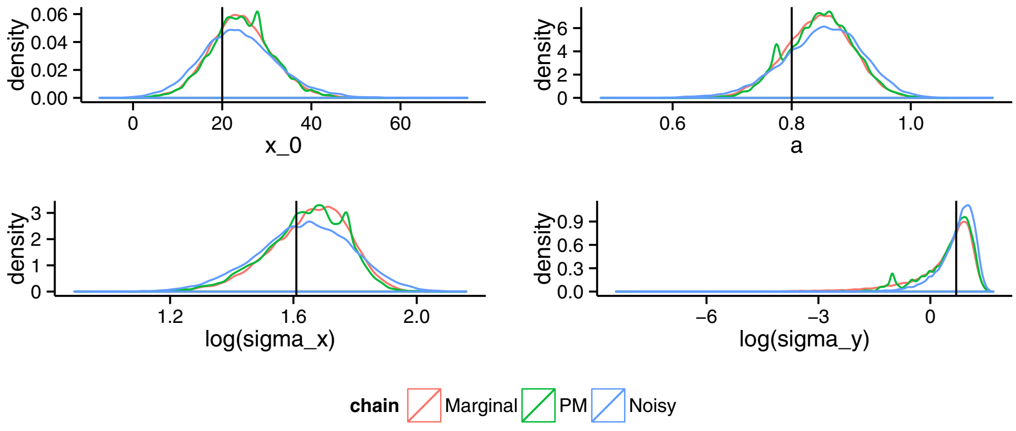

Fig. 26 Estimated densities using the marginal, pseudo-marginal and noisy chains for the 4 parameters when $N = 500$. Vertical lines indicate the real values

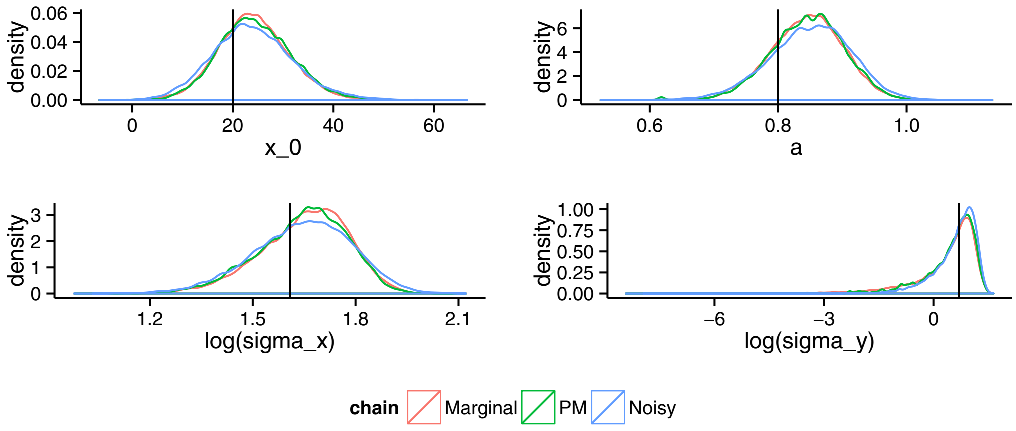

Fig. 27 Estimated densities using the marginal, pseudo-marginal and noisy chains for the 4 parameters when $N = 1,000$. Vertical lines indicate the real values
